# Technology-Assisted Motor-Cognitive Training Among Older Adults: Rapid Systematic Review of Randomized Controlled Trials

**DOI:** 10.2196/67250

**Published:** 2025-06-03

**Authors:** Yaqin Li, Yaqian Liu, Angela YM Leung, Jed Montayre

**Affiliations:** 1 School of Nursing Hong Kong Polytechnic University Hong Kong China (Hong Kong)

**Keywords:** technology-assisted, motor-cognitive, performance, older adults, randomized controlled trials

## Abstract

**Background:**

Age-related physiological changes in older adults involve a rapid decline in motor exercise ability; some older adults may also experience difficulties in maintaining focus, memory loss, and a decline in reaction time, which consequently impair their ability to perform dual tasks. Motor-cognitive training (MCT) refers to a blend of motor activity and cognitive training that occurs simultaneously and can assist older adults in enhancing their physical function, cognitive abilities, and dual-task performance. In recent years, the use of technology for delivering MCT has become increasingly popular in research. This has been achieved through various technologies that simplify MCT for older adults.

**Objective:**

This study aimed to systematically examine the feasibility and effectiveness studies on technology-assisted MCT among older adults.

**Methods:**

This rapid review was conducted following the updated PRISMA (Preferred Reporting Items for Systematic Reviews and Meta-Analyses) 2020 standards, and the Synthesis Without Meta-analysis (SWiM) in systematic reviews reporting guideline. Four databases were searched, including CINAHL, Embase, PubMed, and Scopus, from January 2013 to March 2025. Search strategies were constructed based on three main topics: (1) older adults, (2) MCT, and (3) technology. Inclusion criteria followed the population, intervention, comparator, outcome, and study design framework as follows: older adults (population); technology-assisted MCT (intervention); standard treatment control, active control, partial intervention control, placebo control, and dose-response control (comparator); various measures of physical, cognitive, and dual-task performance (outcome); and randomized controlled trials (RCTs) and pilot RCTs (study design). The Cochrane Risk of Bias Tool was applied for quality appraisal of the included studies. The feasibility of the included studies was assessed using completion rates and attrition rates. Descriptive statistics were used to describe the demographic and clinical characteristics of the groups, while narrative methods were used to categorize and synthesize their effectiveness.

**Results:**

In total, 20 studies were included, comprising 16 RCTs and 4 pilot RCTs, most of which were conducted within a 6-week period. Each session typically lasted between 10 and 30 minutes and was held 2 to 3 times per week. Feasibility analysis showed that technology-assisted MCT was generally feasible. While the workload was high, the perceived usability was also high, with a considerable amount of positive feedback and very few reported adverse events. The types of MCT varied in terms of components, duration, and frequency. The majority of studies (18/20, 90%) demonstrated statistically significant improvements in physical, cognitive, and dual-task performance because of technology-assisted MCT.

**Conclusions:**

The feasibility of technology-assisted MCT among older adults was high regardless of the perceived high workload, and most studies showed statistical effectiveness in improving physical, cognitive, and dual-task performance.

**Trial Registration:**

Open Science Foundation (OSF) Registries 10.17605/OSF.IO/5SRCQ; https://osf.io/5srcq

## Introduction

### Background

The United Nations projects that the number of people aged ≥65 years will reach 1.6 billion by 2050, accounting for 16% of the total population [1]. Older adults will experience age-related decline regarding their physical, cognitive, and dual-task reserves and functions [2]. For example, physically, most of them will experience osteoporosis, connective tissue problems [3,4], and muscle fiber loss [5], contributing to dysfunction [6,7]. Cognitively, processing speed demonstrates the earliest decline [8], along with a serious decline in learning ability, attention, visual-spatial capability, and working memory [9-11]. These factors collectively decrease their physical and motor reserves and functions, while cognitive decline will further lead to issues such as memory loss, difficulty maintaining focus, and ultimately slower thinking [12,13]. “Dual tasking” refers to performing 2 tasks simultaneously, typically combining cognitive and motor tasks of varying complexity, such as doing simple arithmetic like addition or subtraction while walking [14,15]. Dual tasks are common in daily life and demand the simultaneous processing of multiple pieces of information and tasks, placing greater strain on physical and cognitive reserves and functions [16,17]. Older adults not only experience a simultaneous reduction in both motor and cognitive reserves and functions, resulting in a gradual or sharp decline in their ability to perform dual tasks, but also reductions in both processing speed, as mentioned earlier, and reaction time [18], which further impairs their ability to perform dual tasks. In the meantime, limited opportunities to perform dual tasks in daily life conversely result in the deterioration of physical, cognitive, and dual-task reserves and functions [19]. These changes result in decreased physical, cognitive, and dual-task reserve and functions in older adults [5-8], and they ultimately impact their activities of daily living (ADLs) [20].

Motor-cognitive training (MCT) is the most typical type of dual task, which combines motor activity (eg, aerobic, strength, and endurance training) with cognitive training (eg, memory and attention training) [21,22]. This approach significantly improves physical, cognitive, and dual-task performance in various populations [23-25]. For instance, a systematic review and meta-analysis of 11 randomized controlled trials (RCTs) with 322 participants showed that MCT improved gait, motor symptoms, and balance in patients with Parkinson disease (PD) [26]. Another review of 13 studies with 584 patients found MCT to be beneficial for balance in patients with multiple sclerosis [27]. In addition, MCT offers synergistic benefits by combining motor and cognitive training, enhancing brain adaptations and cognition [28]. A review of 21 studies with 2221 participants showed small-to-medium improvements in cognitive functions in older adults with cognitive impairment [23]. Synthesized evidence on MCT showed that it improves dual-task performance in patients with PD [29] and positively impacts cognitive and physical functions in individuals with dementia or mild cognitive impairment [30].

In recent years, as technology is becoming increasingly integrated into our daily lives, its use for delivering MCT has also become increasingly popular in research [31]. Technology-assisted intervention involves incorporating technology—such as digital devices, tele-technology, monitoring, and assistive technology—into clinical interventions to assess, monitor, educate, and enhance the effectiveness of an intervention [32]. Technology can address challenges in traditional MCT by refining technological innovations in response to specific needs and creating interactive scenarios [31]. The use of technology can provide participants with standardized and uniform instructions on their interventions, ensuring consistency of MCT [33,34]. Technology can make MCT more feasible, enjoyable, and relaxing for older adults [35-38], providing timely assistance and feedback [39-42], and increasing the likelihood of voluntary participation and long-term adherence [43-46]. Technology enhances the motor activity component of MCT by offering premade demonstrations and real-time environments, reducing the need for trainers and reserved spaces [47,48]. Sensors and interactive technologies can monitor movements in real time, ensuring proper posture and technique [49-51], and record exercise processes for future improvement [52,53]. In addition, technology can expand cognitive training beyond basic tasks, offering diverse and engaging exercises through virtual reality (VR) and exergames, which simulate complex cognitive scenarios [54,55]. Despite these advantages, few reviews have investigated the feasibility and effectiveness of technology-assisted MCT among older adults.

### Objectives

In this study was a rapid systematic review to identify various types of technology-assisted MCT and examine the literature in terms of the feasibility and effectiveness of technology-assisted MCT in older adults.

## Methods

### Overview

This rapid review was conducted in accordance with the updated Preferred Reporting Items for Systematic Reviews and Meta-Analyses (PRISMA) 2020 standards [56], and the Synthesis Without Meta-analysis (SWiM) in systematic reviews reporting guidelines [57]. The PRISMA and SWiM checklists can be found in Multimedia Appendices 1 and 2, respectively. In addition, 2 other guidelines were used: “Rapid Reviews to Strengthen Health Policy and Systems: A Practical Guide” from the Alliance for Health Policy and Systems Research, World Health Organization (WHO) [58], and “Conducting a Rapid Review for Quick Turnaround Knowledge Synthesis” [59,60]. These methodologies were integrated into the process from the search strategy to the presentation of results. This review was registered on the Open Science Framework platform [61]. Details of registration are provided in Multimedia Appendix 3.

### Search Methods

We conducted a rapid systematic search for relevant studies using 4 databases, including CINAHL, Embase, PubMed, and Scopus, focusing on literature from the past decade (from January 2013 to March 2025) [58,60]. To ensure comprehensiveness, we also reached out to the authors of eligible studies without full texts, authors of conference abstracts, and authors of important studies. Our search terms centered on three main topics: (1) older adults, (2) MCT, and (3) technology, by using Medical Subject Headings (MeSH), terms Emtree terms, free-text terms, combinations of these terms, and Boolean operators (eg, and, or, and not). For example, terms like *aged* (MeSH, Emtree), senior*, *elderly* (Emtree) for older adults; *DT*, *concurrent-task*, *cognitive-motor* for MCT; and *technology* (MeSH, Emtree), *virtual reality* (MeSH, Emtree), *exergaming* (MeSH) for technology were applied. Details of our search strategies can be found in Multimedia Appendix 4.

### Inclusion and Exclusion Criteria

Published papers were included in this review according to the inclusion criteria following the population, intervention, comparator, outcome, and study design (PICOS) framework [62]. The exclusion criteria are also provided in the subsequent sections. More details of the inclusion and exclusion criteria for the included studies in this review are in Textbox 1.

**The inclusion and exclusion criteria of the studies included in this review.**

**Inclusion criteria**

- Older adults aged >55 years
- Motor-cognitive training (MCT) delivered using any technology as a whole or part of the intervention
- Standard treatment control, active control, partial intervention control, placebo control, and dose-response control
- A variety of measures related to the physical, cognitive, and dual-task performance
- Randomized controlled trials (RCTs) and pilot RCTs

**Exclusion criteria**

- Adults aged <55 years
- Non-MCT or MCT not delivered using any technology as a whole or part of the intervention
- Other measures not focusing on physical, cognitive, and dual-task performance
- Other non-RCT studies (eg, pre-post studies)

### Inclusion Criteria

Details on inclusion criteria following the PICOS framework are as follows:

For population, we included studies that reported that their participants were older adults, without restrictions based on sociodemographic characteristics such as gender, medical background (clinical or nonclinical population), residence, ethnicity, educational background, or occupational status.

For interventions, technology-assisted MCT was defined as MCT delivered using any technology (including VR, exergames, phones, computers, tele-technology, and other types of technology). We also included studies that used technology-assisted MCT as part of the overall intervention.

For comparators or control, we included studies that compared technology-assisted MCT to treatment. This could be standard treatment control, active control, partial intervention control, placebo control, or dose-response control.

For main outcomes, indicators included a variety of measures related to the physical, cognitive, and dual-task performance of the participants. These measures involved the use of scales, instruments, and sensors to measure physiological metrics of the patient and other tools.

For study design, RCTs and pilot RCTs were included. RCTs use an experimental research design that minimizes bias by randomly assigning participants to intervention and control groups to evaluate the effect of a specific intervention on study outcomes [63]. In contrast, a pilot RCT is a smaller-scale version of an RCT, typically conducted before a formal RCT study, to assess the feasibility of the intervention, the validity of experimental procedures, and the operationalization of data collection, as well as to test the study design [64]. RCTs were included in this review because they provide robust evidence, are better able to minimize bias, and offer high internal validity. Pilot RCTs were also considered because the research question addressed by this rapid systematic review concerns a relatively new intervention that has not been widely studied. Pilot RCTs offer preliminary evidence on feasibility, aligned with the review’s objectives, and provide a reasonable foundation for informing larger RCTs.

### Exclusion Criteria

Exclusion criteria were studies of adults aged <55 years; non-MCT or MCT not delivered using any technology as a whole or part of the intervention; other measures not focusing on physical, cognitive, and dual-task performance; and other non-RCT studies (eg, pre-post studies).

### Screening and Selection

All articles retrieved from the literature search were imported into EndNote (Clarivate), where duplicates were automatically removed. Two researchers (Y Li and Y Liu) managed and selected the literature, following this screening process: first, titles and abstracts were independently screened by the researchers. Full texts were obtained if at least 1 reviewer believed an article met the inclusion criteria. Subsequently, 2 reviewers independently verified the eligibility of references through full-text screening. If disagreements on inclusion arose and could not be resolved through discussion, a third reviewer (JM) was consulted. Reasons for exclusion were documented. The general process of literature search and study selection was described using a flowchart following the updated PRISMA 2020 guidelines [56].

### Quality Appraisal

Two researchers independently reviewed each study for methodological quality and rigor. They used the Cochrane Risk of Bias Tool (version 2.0) [65] for RCTs. In cases of disagreement, a third researcher (JM) was consulted.

### Data Abstraction

Data were independently extracted by 2 researchers using a standardized form, and all processes involved in obtaining and validating data from the original authors were documented. The form included the following information: (1) characteristics of the included papers, such as author, country, year of publication, information regarding participants (sample size, age, and gender), and experimental design; (2) intervention details, including study duration, type of intervention, intervention components (physical and cognitive part), dosages, duration of each session, frequency, intensity, type of control, the content of control, technological device applied in the studies, the form of delivery (online, offline, mixed); (3) information on feasibility, including overall recruitment, retention, attainment, and dropout rates; (4) measures of participants’ physical, cognitive, and dual-task performance. Disagreements during the data extraction process were resolved through discussions with a third-party researcher (JM). All recommendations were incorporated once a consensus was reached. When study data were published in multiple articles, the article with the most detailed information or the largest sample size was selected.

### Data Analysis

The feasibility of the included studies in this review was assessed using completion rates and attrition rates for calculations. In addition, the most-used scales to assess the feasibility of the intervention systems were presented in this review, along with statistical analysis of scores obtained in each study. We reviewed feedback on each intervention from participants in each study and presented several narratives from participants. The frequency of adverse events associated with the interventions was also calculated, along with specific descriptions of those events.

In this study, a rapid systematic review was conducted due to the significant variation among the included studies. These variations encompassed differences in the intervention components (motor and cognitive), settings, the total duration, the duration of each session, frequency, the technologies applied, the time points for outcome measurement, and the outcome indicators. Descriptive statistics were used to describe the demographic and clinical characteristics of the groups, while narrative methods were used to categorize and synthesize the effectiveness.

### Quality Evaluation of the Configuration of the Intervention Components (Motor and Cognitive)

This review analyzed the quality of the configuration for each component, including motor activity and cognitive training parts, and evaluated them according to previous standards or guidelines, to determine if they meet the training standards. The criteria for the quality assessment of each component compiled in this review simply represent the criteria for conducting quality evaluations in this review, to facilitate the following analysis of the effects of all included studies. The aspects and criteria for each inspection were as follows. First, the motor activity aspect: components were considered effective if the study stated that the intervention design was based on exercise guidelines, specific research findings (eg, previous studies or expert opinions), or if the design—including the components, session duration, frequency, and total volume—met WHO recommendations for exercise for older adults [66]. In such cases, interventions were marked with a checkmark. Second, the cognitive training aspect: interventions were considered effective if the study indicated that the intervention design was based on guidelines related to cognitive interventions, or specific research findings (eg, previous studies or expert opinions). In these cases, interventions were marked with a checkmark.

### Evaluation of Effects

In this review, we carefully examined each included study for statistical significance across the following 3 outcome metrics: physical, cognitive, or dual task–related measures. If a study demonstrated statistical significance for a particular outcome (any related outcome indicators in physical, cognitive, or dual-task performance), it was considered valid in that area (eg, physical, cognitive, or dual-task performance). In these cases, if there are some outcome indicators with statistical significance, they were marked with a checkmark.

## Results

### Overview

**Figure 1:**
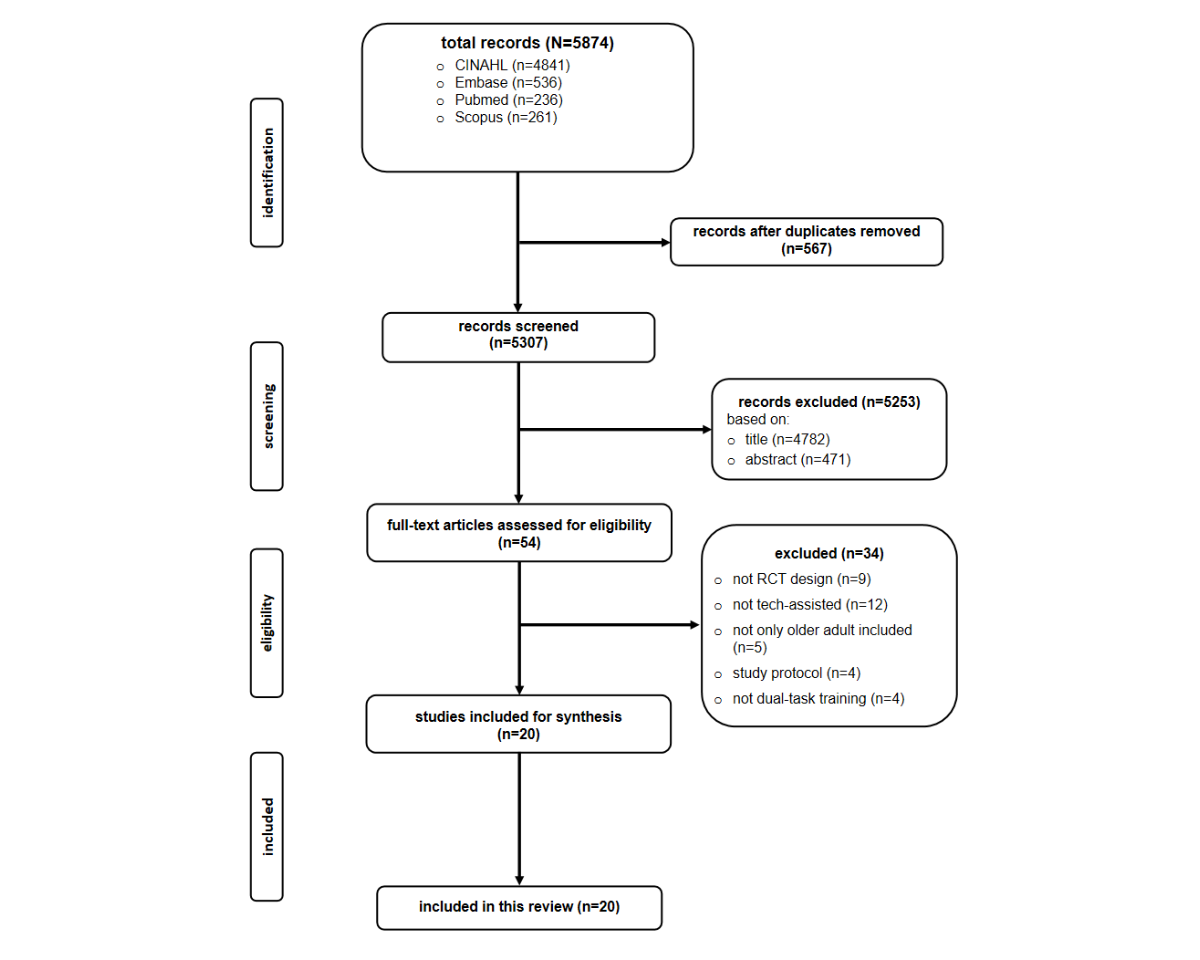
The PRISMA flowchart. RCT: randomized controlled trial.

The literature search yielded 5874 potentially relevant studies. After removing 567 duplicates using EndNote, 5307 were screened by 2 independent researchers. After a thorough evaluation of titles and abstracts, 5253 studies were excluded, of which 4782 were excluded based on their titles, and an additional 471 were removed after reviewing their abstracts, as they did not meet the inclusion criteria of this review. Following this preliminary screening, we conducted a full-text review of the remaining 54 articles, of which 34 were further excluded due to specific reasons outlined in the PRISMA flowchart in Figure 1. Consequently, 20 articles met our inclusion criteria and were included.

### Quality Appraisal

**Figure 2:**
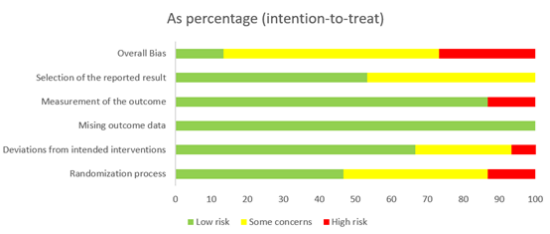
The quality assessment of the included randomized controlled trials (RCTs; n=15; intention-to-treat) as percentages.

**Figure 3:**
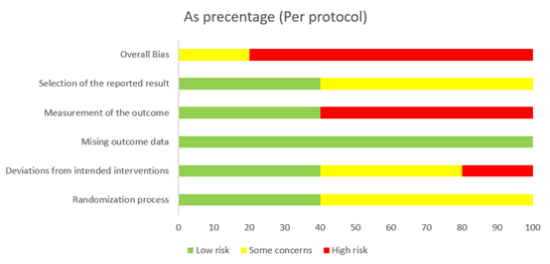
The quality assessment of the included randomized controlled trials (RCTs; n=5; per protocol) as percentages.

Of the 20 RCTs, 8 (40%) had high risk, 10 (50%) had some risk, and only 2 (10%) had low risk. This indicates that most of the trials had some level of risk in their design, resulting in an overall moderate quality. To be specific, there were high levels of risks in 10% (2/20) of studies (studies 8 and 20; the number of studies is consistent with the values presented the tables) and some risks in 45% (9/20) of studies (studies 1, 2, 4, 7, 9, 10, 14, 17, and 18) regarding randomization [67-86]. Regarding deviations from the intended intervention, 10% (2/20) of studies (studies 16 and 18) had a high level of risk, and 30% (6/20) of studies (studies 5, 11, 13, 14, 19, and 20) had some risk. For the measurement of outcomes, 25% (5/20) of studies (studies 5, 8, 13, 17, and 19) showed high risk. Finally, in the selection of reported results, 50% (10/20) of studies (studies 2, 3, 4, 7, 8, 14, 15, 16, 17, and 19) had some risk. The details of quality appraisals of these 20 RCT studies are presented in Figures 2 and 3 and Table 1.

**Table 1:** Risk of bias for included randomized controlled trials (n=20).

Number	Study	Randomization	Deviations from intended intervention	Missing outcome data	Measurement of outcome	Selection of reported result	Overall
1	Menengi et al [67], 2022	Some concerns	Low	Low	Low	Low	Some concerns
2	Jäggi et al [68], 2023	Some concerns	Low	Low	Low	Some concerns	Some concerns
3	Kwan et al [69], 2021	Low	Low	Low	Low	Some concerns	Some concerns
4	Altorfer et al [70], 2021	Some concerns	Low	Low	Low	Some concerns	Some concerns
5	Manser et al [71], 2023	Low	Some concerns	Low	High	Low	High
6	Fishbein et al [72], 2019	Low	Low	Low	Low	Low	Low
7	Kannan et al [73], 2019	Some concerns	Low	Low	Low	Some concerns	Some concerns
8	Uematsu et al [74], 2023	High	Low	Low	High	Some concerns	High
9	Liao et al [75], 2019	Some concerns	Low	Low	Low	Low	Some concerns
10	Schoene et al [76], 2015	Some concerns	Low	Low	Low	Low	Some concerns
11	Forte et al [77], 2023	Low	Some concerns	Low	Low	Low	Some concerns
12	Pelosin et al [78], 2021	Low	Low	Low	Low	Low	Low
13	Eggenberger et al [79], 2015	Low	Some concerns	Low	High	Low	High
14	Delbroek et al [80], 2017	Some concerns	Some concerns	Low	Low	Some concerns	Some concerns
15	Hagovska and Nagyova[81], 2017	Low	Low	Low	Low	Some concerns	Some concerns
16	Park et al [82], 2020	Low	High	Low	Low	Some concerns	High
17	Villa-Sánchez et al [83], 2023	Some concerns	Low	Low	High	Some concerns	High
18	Buele et al [84], 2024	Some concerns	High	Low	Low	Low	High
19	Zak et al [85], 2024	Low	Some concerns	Low	High	Some concerns	High
20	Kwan et al [86], 2024	High	Some concerns	Low	Low	Low	High

### Data Characteristics of Included Studies

A total of 20 RCTs were included in this review, of which only 4 (20%) were pilot RCTs (studies 1, 2, 4, and 5); the remaining 16 (80%) studies were RCTs. Only 5% (1/20) of study focused solely on feasibility (study 17), while 20% (4/20) of studies examined both feasibility and effects (studies 2, 3, 4, and 5). The remaining 75% (15/20) of studies exclusively investigated the effectiveness of their research subject (eg, improvement of gait performance, balance performance, and fall prevention). These papers were published between 2015 and 2024, with a notable uptick in 2023 (6/20, 30%), and were published in 13 different countries, namely, Switzerland (4/20, 20%), Italy (3/20, 15%), China (3/20, 15%), and other countries (10/20, 50%), including the United States, Korea, Ecuador, Poland, Turkey, Israel, Japan, Australia, Belgium, and Slovak Republic. These works were disseminated across 17 distinct journals, with the highest number of publications found in *Frontiers in Aging Neuroscience* (4/20,20%). In total, 10% (2/20) of studies applied co-design (studies 5 and 20) while others reported no co-design elements, and only 5% (1/20) of study reported study fidelity (study 20). Co-design is a design methodology that emphasizes collaboration among multiple parties, particularly stakeholders such as end users, researchers, and others [87]. It encourages active participation throughout the design process by communication, creative input, sharing insights, and testing new ideas, to ensure the outcome better aligns with the needs, desires, and real-world contexts of the stakeholders (eg, development of a new exergame). Half of the included studies (studies 3, 6-9, 12-15, and 18; 10/20, 50%; all RCTs) did not perform sample size calculations. More details are provided in the summary table in Multimedia Appendix 5.

This review analyzed data from 20 studies, encompassing 1197 participants. The population size in these studies ranged from a minimum of 16 to a maximum of 293 participants, with an average of 59.9 (SD 4075.5) participants per study. Although all the included studies stated they would focus on older adults, and most participants were aged ≥60 years, only 1 (5%) study included individuals aged ≥50 years in their study (study 7).

### Study Designs of Included Studies

The following are some of the basic characteristics of the included studies.

#### Inclusion and Exclusion Criteria of Participants in the Included Studies

Most of the included studies stipulated that participants should be older adults; however, the age of participants stipulated in these studies ranged from >50 years (studies 2, 4, and 5), to ≥70 years (studies 10, 13, and 19). The participants’ physical conditions differed, encompassing healthy and nondependent community-dwelling older adults (studies 3, 8, 10, 11, 13, and 17), individuals with mild cognitive impairment (studies 9, 16, 18, and 20), those diagnosed with idiopathic PD (studies 2 and 12), stroke patients (studies 6 and 7), hospitalized older adults (studies 4 and 14), and patients with Alzheimer (study 1).

At baseline, many studies screened participants using cognitive assessment tools, such as the Brief Mental State Examination (studies 1, 2, 4, 16, 17, and 19), or the Montreal Cognitive Assessment (studies 3, 9, 14, 18, 20). In addition, regarding physical abilities, standing or walking for a specified duration was another criterion. These included standing for 3 minutes (studies 2 and 4), standing unassisted or assisted for 5 minutes (studies 7 and 12), and standing for 10 minutes (study 5). Additional criteria encompassed walking with or without a walker (study 10), walking unassisted or assisted for 10 meters (studies 6, 9, and 14), and walking with or without assistance for 20 meters (study 13).

Independence in performing daily activities was also a common requirement (studies 5, 8, 9, 10, 11, and 16). Furthermore, participants were expected to possess the ability to provide informed consent (studies 2, 4, 13, 16, and 18), and adequate communication skills (studies 1, 7, and 16). Finally, some studies also had criteria related to a stable medication regimen or a history of specific treatments (studies 1, 2, and 12) and if the participant had a history of falls (studies 8 and 12).

#### Exclusion Criteria Applied in the Studies

The most-mentioned exclusion criteria among included studies were cognitive impairment, such as dementia (studies 3, 7, 9, 10, 12-15, 18, and 20), or significant cognitive deficits in potential participants. In addition, the inability to comprehend instructions or effectively communicate (often due to language barriers or sensory impairments), often led to exclusion in some cases (studies 1-7, 12, 14-16, and 18-20). Moreover, individuals with unstable or acute medical conditions, including major mental illnesses, were also typically ineligible (studies 1, 2, 4, 5, 9, 10, 12, 15, 16, 18, and 19). Finally, participants who had recently undergone major surgery or had acute orthopedic conditions were generally excluded (study 14). The history of falls also served as an exclusion criterion in certain studies (study 6).

### Technologies Applied in the Studies and Configuration of the Intervention Components (Motor and Cognitive) of MCT

In this review, we reviewed and confirmed that all the included studies used simultaneous MCT, except the study by Buele et al [84]. Notably, we found that MCT showed significant variation in the configuration of components (motor activity and cognitive training), as well as in the duration and frequency of each session of each component. Among the included types of MCT, some were more focused on motor activity interventions involving simple cognitive tasks such as performing addition and subtraction while engaging in specific-intensity, multicomponent, structured motor exercise (eg, aerobic, strength, and balance training). Others were more cognitively focused, structured within a broad cognitive exercise framework, where participants performed simple gross motor activities such as moving their legs back and forth. Also, there were no golden-standard guidelines on how to structure MCT interventions to simultaneously improve physical, cognitive, and dual-task performance. More details are provided in Multimedia Appendix 5.

In Table 2, we briefly describe the components of the motor activities and cognitive training interventions for each MCT, noting whether technology was used in these 2 components, and specifying the technologies used in either 1 part or the overall MCT. We also report whether either intervention component (physical and cognitive) was developed based on ADLs (eg, dressing, eating, or mobility), or instrumental ADLs (IADLs; eg, shopping, housekeeping, or laundry). In Table 2, for the motor activity part, studies 2, 4, and 5 were developed based on ADLs, while study 16 was based on IADLs. Studies 3, 6, 12, 15, 18, and 20 did not use technology for motor activities. Among these, studies 3, 6, and 12 used treadmills, whereas study 15 focused on stamina training. Regarding cognitive training, studies 2, 4, 5, and 15 were based on ADLs, whereas studies 3, 9, 16, 18, and 20 were based on IADLs. Only study 7 did not incorporate technology. The following technologies frequently mentioned among the included studies, including the Dividat Senso (3/20, 15%), the Wii Fit Games (2/20, 10%), exergames besides Wii Fit Games (5/20, 25%), VR noted in various forms (8/20, 40%), and 2 other technologies in 2 (10%) studies. In this study, there were 7 active controls (studies 3, 9, 11, 15, 16, 18, and 19), 5 (25%) studies used standard treatment control as a control group (studies 1, 2, 4, 5, and 20), 4 placebo controls (studies 8, 10, 14, and 17), 3 partial intervention control (studies 6, 7, and 13), and 1 dose-response control (study 12). In addition, regarding the implementation of MCT, 12 (60%) out of 20 studies reported their intervention settings. Specifically, 4 (20%) out of 20 studies (studies 3, 18, 19, and 20) in community settings, 3 (15%) studies (studies 1, 5, and 10) implemented interventions at home, 3 (15%) studies (studies 2, 4, and 7) in clinical rehabilitation settings, 1 (5%) study (study 15) in outpatient settings, and 1 (5%) study (study 14) in a residential care center. More details are provided in Table 2.

**Table 2:** Intervention components and how technologies assisted the interventions.

Number	Study	Physical	Cognitive	How to cooperate	Control group	Settings
1	Menengi et al [67], 2022	Chair-based exercises	Cognitive training facilitated through a computer or tablet	Online supervision (Zoom)	Standard treatment control	Home-based
2	Jäggi et al [68], 2023	Regular rehabilitation and exercises included in the Dividat Senso^a^	Exercises included in the Dividat Senso^a^	The Dividat Senso	Standard treatment control	In an inpatient rehabilitation setting
3	Kwan et al [69], 2021	Cycling on an ergometer^b^	Cognitive training on VR^c^	VR platform	Active control	In community
4	Altorfer et al [70], 2021	Regular rehabilitation therapy and exercises included in the Dividat Senso^a^	Exercises included in the Dividat Senso^a^	The Dividat Senso	Standard treatment control	In a geriatric inpatient rehabilitation setting
5	Manser et al [71], 2023	Exercise based on the brain-IT system (heart rate variability-induced resonance respiration) in combination with the exercises included in the Dividat Senso^a^	Combination of cognitive training^a^ based on the brain-IT system and the Dividat Senso	The brain-IT system and the Dividat Senso	Standard treatment control	At home
6	Fishbein et al [72], 2019	Treadmill^b^	The SeeMee system	The SeeMee system	Partial intervention control	—^d^
7	Kannan et al [73], 2019	Body movement, balance games	Addition, subtraction, and multiplication^b^	Wii Fit, balance board	Partial intervention control	In clinical stroke rehabilitation settings
8	Uematsu et al [74], 2023	Balance training	Cognitive training included in Wii Fit Games	Wii Fit	Placebo control	—
9	Liao et al [75], 2019	The physical regimen comprised resistance, aerobic, and balance exercises aligned with the American College of Sports Medicine standards for older adults	Activities like reciting poems while walking, naming flowers and animals while navigating obstacles, and solving math problems during resistance exercises^c^	VR	Active control	—
10	Schoene et al [76], 2015	4 specific stepping games, balance training	Stepping training targeted cognitive functions of fall prevention in older adults	Electronic pedals, computer interface, and television screen	Placebo control	At home
11	Forte et al [77], 2023	Gross motor coordination (upper and lower body movements designed to enhance both static and dynamic balance)	Stimulus-response cognitive tasks generated by an device (Witty-SEM)	Witty-SEM	Active control	—
12	Pelosin et al [78], 2021	Using a Kinect camera to capture participants’ foot movements on a treadmill, the system integrated these movements into a computer-generated virtual environment displayed on a screen, and participants were required to avoid virtual obstacles^b^	Various cognitive domains, such as executive functions, attention, working memory, and visual processing	A TT+VR system (V-TIME), a Kinect camera	Dose-response control	—
13	Eggenberger et al [79], 2015	The training was designed following current physical fitness and fall prevention recommendations for older adults, focusing on aerobic endurance exercises	Cognitive training	A VR video game, DANCE	Partial intervention control	—
14	Delbroek et al [80], 2017	Physical training in 9 exercises designed to improve balance, weight-bearing, memory, attention, and dual-tasking abilities	Cognitive training in 9 exercises designed to improve balance, weight-bearing, memory, attention, and dual-tasking abilities	The BioRescue	Placebo control	In a residential care center
15	Hagovska and Nagyova [81], 2017	Physical training^b^	Cognifit^a^	Cognifit	Active control	In an outpatient psychiatric clinic of the Highly Specialized Geriatric Institute
16	Park et al [82], 2020	A device with software using VR for activities such as driving, bathing, cooking, and shopping^c^	This setup facilitated training in attention, memory, problem-solving, and executive functions^c^	The MOTOcog system	Active control	—
17	Villa-Sánchez et al [83], 2023	Walking on carpet	Counting backward	Sensing carpet	Placebo control	—
18	Buele et al [84], 2024	Balance exercises (eg, walking in a straight line, squats, brisk walking, going up and down stairs, and social dancing individually and in pairs)^b^	Collective social interaction activities, and an immersive VR-based system that simulates a task of searching for ingredients in a kitchen cupboard^c^	VR	Active control	Community
19	Zak et al [85], 2024	Walking, walking with putting a ball between the hands, and walking with tossing a ball	Cognitive exercises, talking, adding up numbers, subtracting numbers, repeating phrases, reciting a word chain, and identifying objects	VR	Active control	Community
20	Kwan et al [86], 2024	Biking^b^	The VR game contains 8 different themes: orientation, finding a bus stop, reporting lost items, finding a supermarket, grocery shopping, cooking, finding a travel hot spot, and bird watching^c^	VR	Standard treatment control	Community

^a^Interventions developed based on activities of daily living.

^b^Nontechnologically supported components.

^c^Interventions developed based on instrumental activities of daily living.

^d^Not available.

### Duration of Total Experiments and Each Session and Intervention Frequency

The intervention period varied from 2 weeks to 6 months, with many studies lasting 6 weeks (6/20, 30%), 2 to 4 weeks (4/20, 20%), 8 weeks (3/20, 15%), and 12 weeks (3/20, 15%). Each intervention session lasted between 10 and 30 minutes (12/20, 60%), followed by 30 minutes (4/20, 20%), 40 to 60 minutes (3/20, 15%), the other studies did not specify the exact times for the interventions. Regarding frequency, most interventions occurred 2 to 3 days per week (8/20, 40%), followed by 4 to 5 days per week (4/20, 20%). More details can be found in Multimedia Appendix 5.

### Feasibility Analysis

#### Feasibility, Acceptability, and Adherence

Regarding feasibility, acceptability, and adherence, most included studies have shown high completion and low attrition rates (studies 1, 2, 4, 6-8, 10, and 13-17), indicating high participant engagement and intervention suitability.

#### The NASA Task Load Index and the System Usability Scale

The NASA Task Load Index (TLX) is a widely used subjective multidimensional assessment tool that rates perceived workload to evaluate the effectiveness or other aspects of the performance of a task, system, or team. The scale has a total score of 100, with workload score values of 0 to 9 as low, 10 to 29 as moderate, 30 to 49 as slightly high, 50 to 79 as high, and 80 to 100 as very high [88]. Two articles in this review applied the NASA-TLX (studies 2 and 4), with the highest NASA-TLX mean score of 56.2 (study 2) and the lowest of 45.5 (study 4), indicating high workloads.

The System Usability Scale (SUS) is a commonly used tool to analyze the perceived usability of a system, product, or service, scored on a scale of 0 to 100, where the higher the score, the better the usability [89]. Total 3 (15%) of the 20 included studies measured SUS (studies 2, 4, and 5), with mean SUS scores ranging from 71.7 to 83.6, providing better information on overall participant satisfaction and user-perceived usability of the system.

#### Positive Feedback

Positive feedback from participants and medical staff (studies 1 and 14) included the following: “the caregivers stated that they agreed 100% with the expressions-my patient was satisfied with the online exercise treatment” and “I was satisfied with the online exercise treatment” from study 1 and “Interviews with participants from the intervention group showed that they found the program useful for their concentration, memory, and balance, according to the results of the IMI, which resulted in high compliance. They scored the program as very interesting and pleasant to do and perceived their performance of the different exercises as good to very good” from study 14. This demonstrated acceptance and satisfaction with the interventions. These similarities suggest that the interventions were generally well-received, feasible to implement, and safe for participants from different study backgrounds.

### Adverse Events

The results from the included studies consistently highlight the absence of significant adverse events throughout the study period across studies as follows: most of the studies reported no dropouts or adverse events linked to the exercise treatment (studies 1, 2, 4, and 6-17). Among these studies, 20% (4/20) of minor adverse events were noted; however, all evidence suggests that there is no direct link between these adverse events and the intervention. They were “minor technical issues, such as video sound and connection problems, were reported but did not impede the sessions, and caregivers expressed high levels of satisfaction” (study 1); “most participants did not experience symptoms of VR sickness, while two individuals in the control group withdrew due to moderate joint and muscle pain” (study 3); and “specifically falls resulting in bruises, were noted in the intervention group, importantly, these events were unrelated to the ‘Brain-IT’ training” (study 5). Overall, the prevalence of adverse events in these included studies underscores the safety and minimal adverse effects associated with the interventions examined in the review.

### Quality Evaluation of the Configuration of the Intervention Components (Motor and Cognitive)

On the basis of the quality evaluation criteria mentioned in the Quality Evaluations of the Configuration of the Intervention Components (Motor and Cognitive) subsection in the Methods section, 6 of 20 (30%) studies met the standards for motor activity design (studies 5, 6, 9, 11, 13, and 19). In total, 12 of 20 (60%) studies were qualified for cognitive training design (studies 2-5, 10-12, 14-16, 19, and 20). However, only 3 of 20 (15%) studies qualified in both aspects (studies 5, 11, and 19). Details are provided in Table 3.

**Table 3:** The quality assessment of intervention components.

Number	Study	Physical	Cognitive
1	Menengi et al [67], 2022		
2	Jäggi et al [68], 2023		✓
3	Kwan et al [69], 2021		✓
4	Altorfer et al [70], 2021		✓
5	Manser et al [71], 2023	✓	✓
6	Fishbein et al [72], 2019	✓	
7	Kannan et al [73], 2019		
8	Uematsu et al [74], 2023		
9	Liao et al [75], 2019	✓	
10	Schoene et al [76], 2015		✓
11	Forte et al [77], 2023	✓	✓
12	Pelosin et al [78], 2021		✓
13	Eggenberger et al [79], 2015	✓	
14	Delbroek et al [80], 2017		✓
15	Hagovska and Nagyova [81], 2017		✓
16	Park et al [82], 2020		✓
17	Villa-Sánchez et al [83], 2023		
18	Buele et al [84], 2024		
19	Zak et al [85], 2024	✓	✓
20	Kwan et al [86], 2024		✓

### Effects of Interventions and Study Characteristics

Except for 10% (2/20) studies (studies 5 and 17), nearly all studies reported the effects of the intervention, including in physical, cognitive, and dual-task performance, in at least 1 or 2 aspects. The physical and cognitive quality evaluations in Table 4 are derived from the assessments presented in Table 3. More details are provided in Table 4, which is a summary of whether there were any statistically significant outcome indicators in each 3 domains (“yes,” “no,” or “was not tested in this domain”), and Multimedia Appendix 6, which provides the details of statistically significant outcome indicators in the 3 domains.

**Table 4:** The assessment of outcome indicators.

Number	Study	Physical (quality)^a^	Physical (results)^b^	Cognitive (quality)^a^	Cognitive (results)^b^	Dual-task (results)^c^
1	Menengi et al [67], 2022	X^d^	✓^d^	X^e^	✓^e^	—
2	Jäggi et al [68], 2023^f^	X^d^	✓^d^	✓	✓	X
3	Kwan et al [69], 2021^g^	X^d^	✓^d^	✓	✓	—
4	Altorfer et al [70], 2021^f^	X^h^	X^h^	✓	✓	✓
5	Manser et al [71], 2023^f^	✓^i^	X^i^	✓^j^	X^j^	—
6	Fishbein et al [72], 2019	✓^k^	✓^k^	X^l^	—	—
7	Kannan et al [73], 2019	X^d^	✓^d^	X^e^	✓^e^	X
8	Uematsu et al [74], 2023	X^d^	✓^d^	X^l^	—	—
9	Liao et al [75], 2019^g^	✓^k^	✓^k^	X^e^	✓^e^	✓
10	Schoene et al [76], 2015	X^h^	X^h^	✓	✓	—
11	Forte et al [77], 2023	✓^k^	✓^k^	✓	✓	—
12	Pelosin et al [78], 2021	X^h^	X^h^	✓	✓	—
13	Eggenberger et al [79], 2015	✓^k^	✓^k^	X^l^	—	✓
14	Delbroek et al [80], 2017	X^h^	X^h^	✓	✓	X
15	Hagovska and Nagyova [81], 2017^f^	X^d^	✓^d^	✓	✓	—
16	Park et al [82], 2020^g^	X^h^	—^h^	✓	✓	—
17	Villa-Sánchez et al [83], 2023	X^h^	X^h^	X^l^	X^l^	X
18	Buele et al [84], 2024	X^h^	—^h^	X^e^	✓^e^	—
19	Zak et al [85], 2024	✓^k^	✓^k^	✓^j^	X^j^	—
20	Kwan et al [86], 2024	X^d^	✓^d^	✓	✓	—

^a^In the physical (quality) and cognitive (quality) columns, “✓” means met the criteria, “X” means did not meet the criteria, and “—” means not measured.

^b^In the physical (results) and cognitive (results) columns, “✓” means met statistical significance, “X” means did not meet statistical significances, and “—” mean not measured.

^c^In the dual-task (results) column, “✓” means effective in dual-task performance, “X” means ineffective in dual-task performance, and “—” mean not measured.

^d^Consistency of design and results in the motor activity designation did not meet the previous training standards, guidelines for motor activity design, but had motor effects.

^e^Consistency of design and results in the cognitive training designation did not meet the previous training standards, guidelines of cognitive design, but had cognitive effects.

^f^Interventions developed based on activities of daily living.

^g^Interventions developed based on instrumental activities of daily living.

^h^Consistency of design and results in the motor activity designation did not meet the previous training standards, guidelines of motor design, and was without motor effects.

^i^Consistency of design and results in the motor activity designation met the previous training standards, guidelines of motor activity design, but without motor effects.

^j^Consistency of design and results in the cognitive training designation met the previous training standards, guidelines of cognitive training design, but without cognitive effects.

^k^Consistency of design and results in the motor activity designation met the previous training standards, guidelines of motor activity design, and received statistically significant outcomes.

^l^Consistency of design and results in the cognitive training designation did not meet the previous training standards, guidelines of cognitive design, and was without cognitive effects.

### Effects on Physical Performance

This part reported physical performance across multiple studies that showed effectiveness (studies 1-3, 6-9, 11, 13, 15, 19, and 20). Among the physical function-related outcome indicators demonstrating statistical significance, the most commonly applied indicators, including the 5 times sit to stand test (studies 1, 2, and 11), walking speed or time (studies 6, 11, and 13), the timed up and go test (studies 1 and 11), ADL (studies 1 and 15), the Short Physical Performance Battery (studies 2 and 13), gait performance (studies 9 and 13), and fall-related metrics (studies 10 and 13).

### Effects on Cognitive Performance

Results in cognitive function measurements were also reported as effective in some studies (studies 1-4, 7, 9-12, 14-16, 18, and 20). Among the cognitive outcome indicators demonstrating statistical significance, the most common ones included the Montreal Cognitive Assessment (studies 3, 11, 16, 18, and 20), the trail making test parts A and B (studies 9, 15, and 16), the go or no-go test (studies 2 and 4), and visuospatial ability (studies 10 and 12).

### Effectiveness on Dual-Task Performance

Dual-task performance was assessed in only 35% (7/20) of the studies (studies 2, 4, 7, 9, 13, 14, and 17), while only 15% (3/20) of them (studies 4, 9, and 13) showed the effectiveness of the intervention in dual-task performance. Of these 7 studies, there were 2 studies (studies 2 and 4) that were pilot RCTs, while the others were full RCTs.

## Discussion

### General Characteristics of the Included Studies

This is the first systematic review specifically assessing the feasibility and effectiveness of technology-assisted MCT in older adults with various physical conditions. Upon review, there were only 20 RCT studies of technology-assisted MCT interventions. Although interest has been growing yearly, and an uptick in 2023 was captured (6/20, 30%), the number of studies remains relatively sparse. Most studies included in this review used exergame technology to deliver MCT (including systems such as Dividat Senso, Wii Fit Games, and other exergames; 10/20, 50%), and this was closely followed by the use of VR technology (8/20, 40%). Only 5% (1/20) of studies used remote intervention design, and nearly half (10/20, 50%) focused on fall prevention, walking performance, balance, or gait performance. Moreover, co-design was not widely applied (2/20, 10%), despite its importance for boosting user engagement, system usability, user experience [90], and potentially increasing the effectiveness of the training [91,92]. Finally, the sample sizes were generally small, for both the total number of participants and the average number per study. In the meantime, proper sample size estimation methods were only applied in half (10/20, 50%) of the included studies. The average number of participants was <60 [93] and was not only typically seen in all pilot RCTs (studies 1, 2, 4, and 5), but most of the RCTs (studies 3, 6-9, 11, 14, and 16-18).

There are some positive aspects to highlight. This review includes a relatively representative sample involving both healthy and older adults with chronic conditions. Participants were selected based on their physical and cognitive functioning to ensure a proper intervention basis for MCT (eg, sufficient physical, cognitive functioning, communication skills, while excluding participants with severe cognitive or mobility impairments, sensory deficits, acute illnesses, unstable medical conditions, recent major surgeries, or a significant history of falls). The most common total experiment duration was 6 weeks, each session typically lasted 10 to 30 minutes, and they took place 2 to 3 times per week. In experiments, shorter durations with higher frequency effectively avoid participant fatigue, which may also have the potential to impact performance and response [94], reduce attrition, maintain compliance [95,96], and lower the overall cost of the experiment. In addition, the primary outcome of this review demonstrated excellent feasibility, acceptability, and adherence. Although the perceived workload was average to high, the perceived usability of the systems was also high. Furthermore, most studies received positive feedback with few negative events. Overall, the data suggest that the feasibility of this technology-assisted MCT intervention is high, with minimal prevalence of adverse events.

### Relationship Between the Varied Effects and the Different Configurations (Motor and Cognitive Components) of the Technology-Assisted MCT

As mentioned in the introduction, MCT has proven effective in improving physical, cognitive, and dual-task performance among older adults [97]. However, the effectiveness of MCT varied across studies. For example, it varied in exercise effectiveness among older adults [98], gait and balance effectiveness varied among older adults with cognitive impairments [23], cognitive function effectiveness varied among individuals with various clinical conditions [99], and balance-oriented motor activity, cognitive training, and dual-task performance enhancement through performance-related interventions varied among normal older adults [100]. To achieve these benefits, training interventions should have increased difficulty, appropriate intensity, sufficient duration, task specificity, and variable task prioritization [100]. In addition, after carefully examining and cross-checking the effects from each included study of their MCT delivered via various technologies (eg, exergames, VR, and other technologies), we found no significant results. On this basis, we can see that the differences in effects may be blamed on the various configurations of MCT, including motor and cognitive components.

In this review, all the studies included were interpretable except for study 5, which showed inconsistent results. This may be attributed to the small sample size in this study (16/20, 80%) among all the included studies, the older aged participants (mean age 79.9 years in the intervention group and 73.7 years in the control group), and the occurrence of adverse events (3 slight adverse events happened in the intervention group, such as falls in homes with bruises, but no more serious injuries) weakened the effectiveness of the interventions. For control groups, there were 7 active controls, 25% (5/20) of studies used standard treatment as a control, 4 used placebo controls, 3 used partial intervention controls, and 1 used a dose-response control. Except for studies that included standard treatment as a control or a placebo control, the remaining studies with other kinds of control groups can be compared to noninferior experiment designs. As a result, there may be an ability to account for the greater effectiveness in the experimental group than the control group.

This review found that physical designs that follow the recommended requirements (eg, for volume, duration, frequency) for motor activity training based on previous research and WHO guidelines [14,66] form a basis to receive exercise benefits. Cognitive gain, on the other hand, depends not only on physical design, but also the cognitive design and whether the participant has an associated cognitive impairment. Moreover, it is possible that physical designs meeting the above criteria are a basis for obtaining beneficial performance in dual tasks. Regarding the design of motor activity interventions, 5 of 20 (25%) studies, (partial intervention control: studies 6 and 13; active control: studies 9, 11, and 19) met the criteria for motor activity, and all these 5 physical measures showed statistical significance. For cognitive design, studies 6 and 13 did not meet the criteria and did not assess cognitive performance. The cognitive intervention in study 11 met the criteria and showed statistically significant cognitive outcomes. Study 9 did not meet the criteria for cognitive training but demonstrated statistical significance in cognitive outcome measures. This may be attributed to the achieved level of physical activity in study 9, which was based on neuroplasticity theory [101], positively influencing cognitive function. Conversely, study 19 met the criteria for cognitive training, but did not show significant cognitive improvements. Upon reviewing study 19, which focused on healthy older adults whose score on the Brief Mental State Examination was >23, its absence of cognitive impairment potentially explains the lack of statistical significance. Finally, studies 6, 11, and 19 did not measure dual-task effects, while studies 9 and 13 demonstrated statistical significance.

All adults should undertake regular physical activity (a strong recommendation with moderate-certainty evidence) according to the WHO guidelines [66]. In this review, 7 studies (standard treatment control: studies 1, 2, and 20; active control: studies 3 and 15; partial intervention control: study 7; placebo control: study 8) did not meet the criteria for motor activity yet showed physical statistical significance. This discrepancy, along with the WHO recommendations for older adults’ motor activity, may suggest that even though their motor activity did not meet recommended standards, participants still gained exercise benefits. In addition, this finding also reveals that this rule is adaptive to technology-assisted MCT. Among these 8 studies, only 4 (studies 2, 3, 15, and 20) met the criteria for cognitive intervention. However, all studies except for study 8, which did not measure cognitive outcomes, showed significant results. These results also can be explained by the neuroplasticity theory that motor activity can enhance participants’ cognitive performance [101]. In addition, the WHO recommendations for cognitive intervention for older adults with or without cognitive decline [14] support the use of cognitive interventions to prevent cognitive deterioration. Overall, the cognitive intervention designs in these studies yielded statistically significant results, even though only half of these studies (4/20, 20%) met the criteria for cognitive intervention design. Of the 7 studies, only studies 2 and 7 assessed dual-task performance, but neither showed statistical significance.

Although motor activity prompts positive and statistically significant effects as advocated by the WHO, the exact tipping point for achieving physical gains remains unclear. In this review, 7 studies (standard treatment control: study 4; placebo control: studies 10, 14, and 17; active control: studies 16 and 18; dose-response control: study 12) did not meet the criteria for motor activity and did not show any statistically significant physical effects (study 16 did not measure physical effects). More research will be needed to determine the type of motor activity, frequency, session duration, and total volume to start to achieve physical benefits. For cognitive training, 5 studies (studies 4, 10, 12, 14, and 16) met the criteria and showed effects, while study 18, despite it not meeting the criteria, showed statistically significant cognitive gains. Compared to the 5 studies (studies 6, 9, 11, 13, and 19) and 7 studies (studies 1, 2, 3, 7,8, 15, and 20) mentioned earlier, this demonstrates that, in technology-assisted MCT, even if the motor activity does not meet the criteria and does not result in effects, the cognitive component, if met, can still achieve a significant cognitive effect (studies 4, 10, 12, 14, and 16). In addition, these 5 studies (studies 4, 10, 12, 14, and 16) also support the WHO recommendation that “cognitive interventions should be provided to older adults, regardless of their cognitive status, to decrease their deterioration in cognitive functions” [14].

### Relationship Between the Varied Dual-Task Effects and Whether the Motor and Cognitive Components in MCT Were Constructed Based on the ADL and IADL Frameworks

Finally, this review included 4 studies with at least one part of the intervention (motor or cognitive) designed based on an ADL framework (studies 2, 4, 5, and 15), and 5 studies with at least one part of the intervention (motor or cognitive) designed based on an IADL framework (studies 3, 9, 16, 18, and 20). Among these, studies 3, 5, 15, 16, 18, and 20 did not measure dual-task performance. Study 2 measured dual-task performance but found no statistical significance, while studies 4 and 9 measured dual-task performance and reported statistical significance. Examining the motor and cognitive design of studies 4 and 9, we found that although the motor activity in study 4 was substandard and the cognitive design in study 9 was substandard, the intervention design in study 4 followed the ADL framework for both motor and cognitive parts, while study 9 followed the IADL design for the cognitive part. On the basis of these findings and the small sample sizes in this review, we may tentatively hypothesize that it may be possible to improve participants’ dual-task performance or even achieve statistical significance if the intervention constructs the motor and cognitive components of MCT based on the ADL and IADL frameworks.

### Conclusions

This rapid systematic review provides an up-to-date synthesis of evidence for technology-assisted MCT in older adults, addressing a significant knowledge gap in the field. The review found that there is little research on technology-assisted MCT; however, it has been relatively successful in participant inclusion and exclusion for this type of intervention. The review also outlines the most used experimental setups in the included studies. Preliminary results indicate that technology-assisted MCT is highly feasible, with almost no reported adverse events, suggesting it merits further research and replication. In addition, this review analyzed and evaluated the composition and quality of motor activity, cognitive training components, the techniques used, and the effectiveness of these interventions. Most of the included studies showed statistically significant effectiveness. We thoroughly examined the contextual settings of each study (eg, the techniques used and the populations studied) to assess their research outcomes. However, we did not observe any significant differences in the effects of the various techniques applied in these included studies. This review suggests that motor activity and cognitive training that meet the criteria yield statistical significance. However, the exact tipping point for achieving physical or cognitive gains remains unclear. Moreover, given the relatively limited sample sizes in this review, it might be tentatively suggested that to potentially enhance participants’ dual-task performance or even achieve statistical significance, researchers can try to construct the intervention of the motor and cognitive components of MCT based on the ADL and IADL frameworks.

### Recommendation

More research will be needed to determine types of motor and cognitive components, frequency, session duration, total volume, and type of technology to start to achieve physical and cognitive benefits from technology-assisted MCT.

## Appendix

Multimedia Appendix 1 PRISMA 2020 checklist.

Multimedia Appendix 2 Synthesis Without Meta-analysis (SWiM) in systematic reviews checklist.

Multimedia Appendix 3 Open Science Framework Registries technology-assisted motor-cognitive training among older adults; a rapid systematic review of randomized controlled trials.

Multimedia Appendix 4 Search strategy (updated version in March 2025).

Multimedia Appendix 5 Summary table.

Multimedia Appendix 6 Details on statistically significant outcome indicators in each of 3 domains.

## Abbreviations

ADL: activities of daily living
IADL: instrumental activities of daily living
MCT: motor-cognitive training
MeSH: Medical Subject Headings
NASA-TLX: NASA Task Load Index
PD: Parkinson disease
PICOS: population, intervention, comparator, outcome, and study design
PRISMA: Preferred Reporting Items for Systematic Reviews and Meta-Analyses
RCT: randomized controlled trial
SUS: System Usability Scale
SWiM: Synthesis Without Meta-analysis
VR: virtual reality
WHO: World Health Organization

## Data Availability

The data that support the findings of this study are available from the corresponding author upon reasonable request.
