# Supplementary material for: Technology-Assisted Motor-Cognitive Training Among Older Adults: Rapid Systematic Review of Randomized Controlled Trials
Source: JMIR Serious Games. 2025 Jun 3;13:e67250. doi: 10.2196/67250 (PMC12174886; doi:10.2196/67250)
Supplement: Multimedia Appendix 3 [file games_v13i1e67250_app3.pdf]

OSF REGISTRIES Add New My Registrations Help Donate

Technology-assisted motor-cognitive training among older adults: a rapid systematic review

Public registration Updates

Metadata

Landing Page

Intended use
This Generalized Systematic Review Registration Form is intended as a general-purpose registration form. The form is designed to be applicable to reviews across disciplines (i.e., psychology, economics, law, physics, or any other field) and across review types (i.e., scoping review, review of qualitative studies, meta-analysis, or any other type of review). That means that the reviewed records may include research reports as well as archive documents, case law, books, poems, etc. This form, therefore, is a fall-back for more specialized forms and can be used if no specialized form or registration platform is available.

Citation
Van den Akker, O. R., Peters, G. Y., Bakker, C., Carlsson, R., Coles, N. A., Corker, K. S., Feldman, G., , Moreau, D., Nordström, T., Pickering, J. S., Riegelman, A., Topor, M., Veggel, N., Yeung, S., Mellor, D., & Pfeiffer, N. Generalized Systematic Review Registration Form. MetaArXiv.. https://doi.org/g5fj.

Review Methods
In this section, you register the general type, background and goals of your review.

Type of review

A rapid review

-Page, M. J., McKenzie, J. E., Bossuyt, P. M., Boutron, I., Hoffmann, T. C., Mulrow, C. D., Shamseer, L., Tetzlaff, J. M., Akl, E. A., Brennan, S. E., Chou, R., Glanville, J., Grimshaw, J. M., Hróbjartsson, A., Lalu, M. M., Li, T., Loder, E. W., Mayo-Wilson, E., McDonald, S., . . . Moher, D. (2021). The PRISMA 2020 statement: an updated guideline for reporting systematic reviews. bmj, 372, n71. https://doi.org/10.1136/bmj.n71
-Tricco, A. C., Langlois, E., Straus, S. E., & Organization, W. H. (2017). Rapid reviews to strengthen health policy and systems: a practical guide. World Health Organization.
-Hartling, L., Guise, J. M., Hempel, S., Featherstone, R., Mitchell, M. D., Motu'apuaka, M. L., Robinson, K. A., Schoelles, K., Totten, A., Whitlock, E., Wilt, T. J., Anderson, J., Berliner, E., Gozu, A., Kato, E., Paynter, R., & Umscheid, C. A. (2017). Fit for purpose: perspectives on rapid reviews from end-user interviews. Syst Rev, 6(1), 32. https://doi.org/10.1186/s13643-017-0425-7

Review stages

Preparation, Search, Screening, Quality Appraisal, Extraction, Synthesis, and Reporting

Current review stage

Synthesis

Start date

this project started from December 2023, and it is still in progress

End date

December 2024

Background

Technology-assisted motor-cognitive training among older adults: a rapid systematic review

The objective of this review was to systematically assess and examine primary research on the effectiveness of technology assisted motor cognitive training in older adults.

Primary research question(s)

The feasibility and effectiveness of technology assisted motor cognitive training in older adults

Secondary research question(s)

no

Expectations / hypotheses

no

Dependent variable(s) / outcome(s) / main variables

The outcome indicators included a variety of measures related to physical, cognitive,
and dual-task(DT) performance of the participants. These measures involved the use of scales, instruments, sensors to measure physiological metrics of the patient, and other tools.

Independent variable(s) / intervention(s) / treatment(s)

For this study, technology assisted motor cognitive interventions were defined as motor cognitive interventions delivered using any technology (including VR,exergames, phone/computers/tele delivered, and other types of technology). We also included studies that used technology assisted motor cognitive interventions as part of the overall intervention.

We included studies that compare technology assisted motor cognitive interventions to treatment as usual (also referred to as standard or traditional treatment), a waitlist group, or an active control.

Additional variable(s) / covariate(s)

no

Software

endnote, word

Funding

no

Conflicts of interest

no conflict of interests exist

Overlapping authorships

no conflict of interests exist

Search Strategy

In this section, you register your search strategy: the procedures you designed to obtain all (potentially) relevant sources to review (e.g., articles, books, preprints, reports, case law, policy papers, archived documents).

Databases

four databases, including Cinahl, Embase, PubMed, and Scopus, focusing on literature
from the past decade

Interfaces

all four databases were accessed from Institutional Access(the Hong Kong Polytechnic University)

Grey literature

we also reached out to authors of eligible studies without full texts, authors of conference abstracts, and authors of important reviews

Inclusion and exclusion criteria

1. Population
This study aims to demonstrate the effectiveness of technology assisted motor cognitive training for older adults. The eligible study population includes studies that reported their participants were older adults , without restrictions based on sociodemographic characteristics such as gender, medical background (clinical/non clinical population), residence, ethnicity, educational background, and occupational status.
2. Intervention
For this study, technology assisted motor cognitive interventions were defined as motor cognitive interventions delivered using any technology (including VR, exergames, phone/computers/tele delivered, and other types of technology). We also included studies that used technology assisted motor cognitive interventions as part of the overall intervention.
3. Comparator(s)/control
We included studies that compare technology assisted motor cognitive interventions to treatment as usual (also referred to as standard or traditional treatment), a waitlist group, or an active control.
4. Main outcome(s)
The outcome indicators included a variety of measures related to physical, cognitive, and DT performance of the participants. These measures involved the use of scales, instruments, sensors to measure physiological metrics of the patient, and other tools.
5.Study design
Randomized controlled trials, and pilot RCT, and pre post studies were included.

Query strings

Our search terms centered around three main topics: (1) older adults, (2) motor cognitive training, and (3) technology.

Search validation procedure

two researchers searched the database independently, and double-checked the results

Other search strategies

no

Procedures to contact authors

we also reached out to authors of eligible studies without full texts, authors of conference abstracts, and authors of important reviews

We would email the first and corresponding authors initially. If they did not respond, we would send a follow-up email the following week. If there was still no response, we would attempt to contact them directly by calling their office number, which we would obtain from their official affiliation website.

Results of contacting authors

no

Search expiration and repetition

no

Search strategy justification

multiple rounds of discussions were conducted on my decisions about the databases, interfaces, grey literature strategies, query strings, author contact procedures, and search expiration date with supervisor (evidence-based expert), and discussed my decisions about the databases, interfaces, grey literature strategies, query strings with librarians from the PolyU(search specialists)

Miscellaneous search strategy details

no

Screening

In this section, you register your screening procedure: the procedure you designed to eliminate all irrelevant sources from the results of the search strategy (and retain the relevant sources).

Screening stages

Two researchers managed and selected the literatures independently. The screening process proceeded as follows: first, the researchers independently screened titles and abstracts. Full texts were obtained only if at least one reviewer believed an article met the inclusion criteria; then, two reviewers independently verif ied the eligibility of references through full text screening. If disagreements on inclusion arose and could not be resolved through discussion, a third reviewer was consulted. Reasons for exclusion were documented. The general process of literature search and studyselection was described using a flowchart following the PRISMA
guidelines.

Screened fields / blinding

no

Used exclusion criteria

This review included/excluded studies based on the inclusion criteria

Screener instructions

This review screened the studies based on the PICOS principles listed above

No files selected

Screening reliability

Two researchers managed and selected the literatures independently.

Screening reconciliation procedure

If disagreements on inclusion arose and could not be resolved through discussion, a third reviewer was consulted.

Sampling and sample size

we would like to use all sources included through the screening procedure

Screening procedure justification

If disagreements on inclusion arose and could not be resolved through discussion, a third reviewer was consulted. Reasons for

Data management and sharing

no

Miscellaneous screening details

no

Extraction

In this section, you register your plans for data extraction: the procedures you designed to extract the data you are interested in from the included sources. Examples of such data are text fragments, effect sizes, study design characteristics, year of publication, characteristics of measurement instruments, final verdicts and associated penalties in a legal system, company turnovers, sample sizes, or prevalences.

Entities to extract

The form included the following information: (1) characteristics of the included papers, such as authors, country, experimental design, and date of publication; (2) the information of feasibility including overall recruitment, retention, attainment, and dropout rates; (3) intervention details, involving intervention components, dosa ges, duration of each session, frequency, intensity, study duration, type of intervention, type of control, content of control ,

technological device applied in the studies, form of delivery (online/offline/mixed); (4) available assessment tools and measurements used for outcome measurement and outcomes.

Extraction stages

First, quality appraisal of the included studies, then, data extraction according to the form pre-designed.

Data were independently extracted by two researchers using a standardised form, and documented all processes involved in obtaining and validating data from the original authors.

Extractor instructions

no

No files selected

Extractor masking

no

Extraction reliability

Data were independently extracted by two researchers using a standardised form, and documented all processes involved in obtaining and validating data from the original authors. If disagreements on inclusion arose and could not be resolved through discussion, a third reviewer was consulted.

Extraction reconciliation procedure

If disagreements on inclusion arose and could not be resolved through discussion, a third reviewer was consulted.

Extraction procedure justification

we followed all the three guidelines we mentioned above

Data management and sharing

no

Miscellaneous extraction details

no

Synthesis and Quality Assessment

In this section, you register the procedure for the review's synthesis: the procedure you designed to use the data that was extracted from each source to answer your research question(s). This often includes transforming the raw extracted data, verifying validity, applying predefined inference criteria, interpreting results, and presenting results. Additionally, you register procedures you designed to assess bias in individual sources and the synthesis itself.

Planned data transformations

no

Missing data

no

Data validation

no

Quality assessment

The Cochrane Risk of Bias Tool for Randomized Trials (ROB V.2.0) and the Risk o f Bias
In Non randomized Studies of Interventions tool (ROBINS I)I), were applied to evaluat e risk of bias of the RCTs and pre post studies, respectively.

Synthesis plan

no, it's a rapid systematic review

Criteria for conclusions / inference criteria

no

Synthesist blinding

no

Synthesis reliability

Data were independently synthesised by two researchers.If disagreements on inclusion arose and could not be resolved through discussion, a third reviewer was consulted.

Synthesis reconciliation procedure

If disagreements on inclusion arose and could not be resolved through discussion, a third reviewer was consulted.

Publication bias analyses

no

Sensitivity analyses / robustness checks

no

Synthesis procedure justification

If disagreements on inclusion arose and could not be resolved through discussion, a third reviewer was consulted.

Synthesis data management and sharing

no

Miscellaneous synthesis details

no

Copyright © 2011-2024 Center for Open Science | Terms of Use | Privacy Policy | Status | API
TOP Guidelines | Reproducibility Project: Psychology | Reproducibility Project: Cancer Biology
