# Supplementary material for: Technology-Assisted Motor-Cognitive Training Among Older Adults: Rapid Systematic Review of Randomized Controlled Trials
Source: JMIR Serious Games. 2025 Jun 3;13:e67250. doi: 10.2196/67250 (PMC12174886; doi:10.2196/67250)
Supplement: Multimedia Appendix 4 [file games_v13i1e67250_app4.docx]

**Appendix 4 Search strategy**

Database(s): **CINAHL N=4841(2013.1 -2025.3)**

| **#** | **Searches** | **Results** |
| --- | --- | --- |
| 1 | (MH "Aged") | 1,012,216 |
| 2 | TX (senior* OR elderly OR "older adult*" OR "older person*" OR geriatric* OR pensioner* OR "the elderly" OR aging OR older) | 23,975,961 |
| 3 | 1 OR 2 | 24,645,235 |
| 4 | TX (("dual-task*" OR "dual task*") OR ("concurrent-task*" OR "concurrent task*") OR ("concurrent-activit*" OR "concurrent activit*") OR ("cognitive-motor" OR "cognitive motor") OR ("motor-cognitive" OR "motor cognitive") OR ("cognition-motor" OR "cognition motor" ) OR ("motor-cognition" OR "motor cognition") OR ("combined-task*" OR "combined task*") OR ("combined activit*" OR "combined-activit*")) | 70,587 |
| 5 | (MH "Technology") | 2,482,324 |
| 6 | "Exergaming" | 2,168 |
| 7 | (MH "Virtual Reality") | 70,772 |
| 8 | TX ((augment* or mix* or extend* or virtual or computer* or interfac* or tablet* or pad* or laptop* or console* or device* or electro* or phone* or mobile* or app* or mHealth) N2 (software* or game* or exergame* or device* or tech* or assist* or environment* or reali* or stimul* or rehab* or therap* or activ* or interact* or world or play)) | 7,815,453 |
| 9 | TX (“Nintendo” or “Xbox” or “PlayStation” or “Dance Dance Revolution” or Switch or Kinect or PS or DDR or VR or “Eye toy”) | 5,440,809 |
| 10 | 5 OR 6 OR 7 OR 8 OR 9 | 10,337,813 |
| 11 | 4 AND 10 | 13,788 |
| 12 | 3 AND 11 | 16,660 |
| 13 | 3 AND 11  Limiters- Publication Date: 2013.1-2025.3 | 4,841 |

Database(s):**EMBASE N=536 (2013.1 -2025.3)**

| **#** | **Searches** | **Results** |
| --- | --- | --- |
| 1 | aged/ | 4068541 |
| 2 | (senior* or elderly or "older adult*" or "older person*" or geriatric* or pensioner* or "the elderly" or aging or older).mp. [mp=title, abstract, heading word, drug trade name, original title, device manufacturer, drug manufacturer, device trade name, keyword heading word, floating subheading word, candidate term word]. | 2167499 |
| 3 | 1 or 2 | 5206394 |
| 4 | ("dual-task*" or "dual task*") or ("concurrent-task*" or "concurrent task*") or ("concurrent-activit*" or "concurrent activit*") or ("cognitive-motor" or "cognitive motor") or ("motor-cognitive" or "motor cognitive") or ("cognition-motor" or "cognition motor") or ("motor-cognition" or "motor cognition") or ("combined-task*" or "combined task*") or ("combined activit*" or "combined-activit*")).mp. [mp=title, abstract, heading word, drug trade name, original title, device manufacturer, drug manufacturer, device trade name, keyword heading word, floating subheading word, candidate term word] | 17487 |
| 5 | technology/ | 113861 |
| 6 | exergaming/ | 527 |
| 7 | virtual reality/ | 32611 |
| 8 | ((augment* or mix* or extend* or virtual or computer* or interfac* or tablet* or pad* or laptop* or console* or device* or electro* or phone* or mobile* or app* or mHealth) adj2 (software* or game* or exergame* or device* or tech* or assist* or environment* or reali* or stimul* or rehab* or therap* or activ* or interact* or world or play)).mp. [mp=title, abstract, heading word, drug trade name, original title, device manufacturer, drug manufacturer, device trade name, keyword heading word, floating subheading word, candidate term word] | 3424824 |
| 9 | ("Nintendo" or "Xbox" or "PlayStation" or "Dance Dance Revolution" or Switch or Kinect or PS or DDR or VR or "Eye toy").mp. [mp=title, abstract, heading word, drug trade name, original title, device manufacturer, drug manufacturer, device trade name, keyword heading word, floating subheading word, candidate term word] | 305643 |
| 10 | 5 or 6 or 7 or 8 or 9 | 3780255 |
| 11 | 4 and 10 | 2040 |
| 12 | 3 and 11 | 626 |
| 13 | limit 25 to yr="2013 - 2025" | 536 |

Database(s): **PubMed N=236 (2013.1-2025.3)**

| **#** | **Searches** | **Results** |
| --- | --- | --- |
| 1 | aged [MeSH Terms] | [3,666,235](https://pubmed.ncbi.nlm.nih.gov/?term=aged%5bMeSH+Terms%5d&sort=relevance) |
| 2 | ((((((((senior*) OR (elderly)) OR ("older adult*")) OR ("older person*")) OR (geriatric*)) OR (pensioner*)) OR ("the elderly")) OR (aging)) OR (older) | [7,104,360](https://pubmed.ncbi.nlm.nih.gov/?term=((((((((senior*)+OR+(elderly))+OR+() |
| 3 | 1 OR 2 | [7,104,360](https://pubmed.ncbi.nlm.nih.gov/?term=(aged%5bMeSH+Terms%5d)+OR+(((((((((senior*)+OR+(elderly))+OR+() |
| 4 | ("dual-task*" OR "dual task*") OR ("concurrent-task*" OR "concurrent task*") OR ("concurrent-activit*" OR "concurrent activit*") OR ("cognitive-motor" OR "cognitive motor") OR ("motor-cognitive" OR "motor cognitive") OR ("cognition-motor" OR "cognition motor") OR ("motor-cognition" OR "motor cognition") OR ("combined-task*" OR "combined task*") OR ("combined activit*" OR "combined-activit*") | 13,744 |
| 5 | ((Technology[MeSH Terms]) OR (Exergaming[MeSH Terms])) OR (Virtual Reality[MeSH Terms]) | 534,956 |
| 6 | ((augment* OR mix* OR extend* OR virtual OR computer* OR interfac* OR tablet* OR pad* OR laptop* OR console* OR device* OR electro* OR phone* OR mobile* OR app* OR mHealth) NEAR/3 (software* OR game* OR exergame* OR device* OR tech* OR assist* OR environment* OR reali* OR stimul* OR rehab* OR therap* OR activ* OR interact* OR world OR play)) | 131 |
| 7 | ("Nintendo" OR "Xbox" OR "PlayStation" OR "Dance Dance Revolution" OR Switch OR Kinect OR PS OR DDR OR VR OR "Eye toy") | 651,534 |
| 8 | 5 OR 6 OR 7 | 1,174,956 |
| 9 | 4 AND 8 | 781 |
| 10 | 3 AND 9 | 286 |
| 11 | Limiters- Publication Date: 20130101-20250331 | 236 |

Database(s): **SCOPUS N=261 (2013.1-2025.3)**

| **#** | **Searches** | **Results** |
| --- | --- | --- |
| 1 | TITLE-ABS (aged OR senior* OR elderly OR "older adult*" OR "older person*" OR geriatric* OR pensioner* OR "the elderly" OR aging OR older) | 2,678,786 |
| 2 | TITLE-ABS( ( ("dual-task*" OR "dual task*") OR ("concurrent-task*" OR "concurrent task*") OR ("concurrent-activit*" OR "concurrent activit*") OR ("cognitive-motor" OR "cognitive motor") OR ("motor-cognitive" OR "motor cognitive") OR ("cognition-motor" OR "cognition motor") OR ("motor-cognition" OR "motor cognition") OR ("combined-task*" OR "combined task*") OR ("combined activit*" OR "combined-activit*") ) ) | 18,772 |
| 3 | TITLE-ABS (technology OR exergaming OR "virtual reality") | 4,365,833 |
| 4 | TITLE-ABS ( ( augment* OR mix* OR extend* OR virtual OR computer* OR interfac* OR tablet* OR pad* OR laptop* OR console* OR device* OR electro* OR phone* OR mobile* OR app* OR mhealth ) W/ 3 ( software* OR game* OR exergame* OR device* OR tech* OR assist* OR environment* OR reali* OR stimul* OR rehab* OR therap* OR activ* OR interact* OR world OR play ) ) | 95,458 |
| 5 | TITLE-ABS ("Nintendo" OR "Xbox" OR "PlayStation" OR "Dance Dance Revolution" OR switch OR kinect OR ps OR ddr OR vr OR "Eye toy") | 711,749 |
| 6 | 3 OR 4 OR 5 | 5,068,130 |
| 7 | 2 AND 6 | 1,339 |
| 8 | 1 AND 7 | 293 |
| 9 | Limiters - Publication Date: 20130101-20250331 | 261 |
