# Supplementary material for: Technology-Assisted Motor-Cognitive Training Among Older Adults: Rapid Systematic Review of Randomized Controlled Trials
Source: JMIR Serious Games. 2025 Jun 3;13:e67250. doi: 10.2196/67250 (PMC12174886; doi:10.2196/67250)
Supplement: Multimedia Appendix 5 [file games_v13i1e67250_app5.docx]

**Appendix 5-the summary table**

**Table 1- the summary table**

| **Author (Year) Journal (Country)** | **Objective (Intervention, technology, etc.)** | **Study design (Study duration)** | **Feasibility, acceptability, and adherence** | **Inclusion and exclusion criteria（Number of patients）Mean age** | **Adverse events** | **FidelityCo-design** |  |
| --- | --- | --- | --- | --- | --- | --- | --- |
| **1.**Kübra (2022) Clin Neurol Neurosurg (Turkey) | to investigate the effectiveness of a home-based telerehabilitation in AD | an online pilot RCT (-) | Twenty participants were recruited and randomly assigned to the TR and control groups (n = 10 per group); all participants completed the study. There were no dropouts from the study. The caregivers stated that they agreed 100% with the expressions; "My patient was satisfied with the online exercise treatment" and "I was satisfied with the online exercise treatment”. | **Inclusion:** age> 65 years, diagnosis of AD according to the National Institute of Neurological and Communicative Disorders and Stroke and Alzheimer’s Disease (NINCDSADRDA) criteria, Mini-Mental State Examination score of 13–24, Clinical Dementia Rating Scale score 1–2, regular use and stable doses of cholinesterase inhibitors and/or memantine for at least a month, sufficient communication skills to understand the instructions, and living with a caregiver who could use technological equipment  **Exclusion:** dementia types other than AD; pulmonary, neurologic, musculoskeletal or rheumatologic disease that might prevent exercise; unstable medical condition (e.g. uncontrolled diabetes or hypertension, deep vein thrombosis); having routine rehabilitation service from an institution or person; regular exercise habits; and visual or auditory deficits or behavioral problems that would make communication difficult. Participants who had problems in adapting to online applications, and who moved to a different city or home from the time they were included in the study were also planned to be excluded  (20) Telerehabilitation group:77.7 years; Control group: 80.6 years | There were no adverse events related to the exercise treatment. Rarely experienced video, sound, and connection problems did not constraint the sessions | NO/NO |  |
| **2.**Salome (2023) Eur J Med Res (Switzerland) | to test the feasibility and effects of an exergames S-MC in inpatient rehabilitation of PD | a pilot RCT(-) | The attrition rate was 5% (n = 2 participants) with the reasons for drop-out all unrelated to the study. One participant had to stop after the first training session because his health deteriorated drastically, and one participant had to stop the study after the 14th training session due to a new therapy and medication plan that was no longer compatible with the study. As a result, there were no drop-outs due to the intervention and, therefore, the intervention-related attrition rate was 0%. All 19 participants in the intervention group who completed the study had an adherence rate of over 70% for the training sessions and wereincluded in the analysis. The overall adherence rate was 96.5%. Reasons for non-adherence were external medical appointments as well as fatigue, acute pain and severe dyskinesia. Except for the duration of the intervention (more than twice as long) and the conduct of the study with older adults, the exergame intervention was almost identical to the present study | **Inclusion:** (1) prescription for inpatient rehabilitation; (2) age ≥ 50 years; (3) able to score ≥ 20 at the Mini Mental State Examination (MMSE); (4) able to provide a signed informed consent; (5) physically able to stand for at least 3 min without external support (self-report)  **Exclusion:** (1) mobility or cognitive limitations or comorbidities which impaired the ability to use the training games and overall system; (2) conservatively treated osteoporotic fractures; (3) previous or current major psychiatric illness (e.g., schizophrenia, bipolar disorder, recurrent major depressive episodes); (4) history of drugs or alcohol abuse; (5) terminal illness; (6) severe sensory impairments (mainly visual, auditory, color blindness); (7) insufficient knowledge of German to understand the training. Then, demographic and medical data were acquired  (40) 72.4 ± 9.54 years | No adverse events were reported at any time during the study | NO/NO |  |
| **3.**Rick (2021) JMIR Serious Games (HK, China) | to explore the feasibility and effects of VR S-MC in older people with CF | a RCT (from recruitment to completion of follow-up from September to November 2020) | the recruitment rate was acceptable (17/33, 52%). Both groups had a 100% attendance rate. The completion rate of the intervention group (8/9, 89%) was higher than that of the control group (6/8, 75%). Training was terminated for one participant (1/9, 11%) due to minimal VR sickness (Virtual Reality Sickness Questionnaire score=18.3/100). Two participants (2/8, 25%) in the control group withdrew due to moderate leg pain | **Inclusion:** (1) age ≥60 years; (2) community-dwelling, defined as living at home and not having stayed in a long-term care facility (eg, a nursing home) in the past 12 months; and (3) cognitive frailty, defined as the coexistence of MCI and physical frailty without being severe enough to have dementia. MCI was measured according to (1) a Montreal Cognitive Assessment (MoCA) score ≤25 and a Clinical Dementia Rating of 0.5. Frailty status was measured on a scale from prefrail to frail, using the Fried Frailty Phenotype (FFP) scale, which assesses five components of frailty, namely handgrip strength, walking speed, physical activity level, exhaustion, and weight loss with an FFP score of ≥1  **Exclusion:** (1) a diagnosis of dementia, according to the subject’s medical record; (2) probable dementia, as defined by a MoCA score ≤18; or (3) restricted mobility, as defined by a Modified Functional Ambulatory Classification below Category 7 (ie, outdoor walker). This criterion was used because the subject might be unable to complete the motor-training exercises  (17) 74.0 years | The research team terminated the training of 1 participant (11.1%) in the intervention group because they reported repeatedly experiencing mild VR sickness (VRSQ=18.3/100). Although the participant still wanted to continue with the training, the research team decided against this to ensure a high level of safety. In the control group, 6 participants completed the intervention. Two participants withdrew because they reported experiencing a moderate level of leg pain and were unable to participate in the cycling. With regard to adverse outcomes, the vast majority of participants never experienced any symptoms of VR sickness. No other symptoms causing discomfort were reported by the participant | NO/NO |  |
| **4.**Patrizia (2021) Front Aging Neurosci (Switzerland) | to test the feasibility and efficacy of an exergame-based S-MC in geriatric inpatients | a pilot feasibility RCT (during a period of 3 months -January to March 2021) | Thirty-nine persons were included in the study, the attrition rate was 7% (n = 3 participants) and the dropout reasons were all study unrelated. Two participants had to quit prior to the first training and one participant left the clinic after one training due to personal reasons. Consequently, no dropouts occurred for intervention related reasons and, therefore, the intervention-related attrition rate was 0%. The average adherence rate was 99% and the reasons for non- adherence were acute back-pain and acute stomach-ache | **Inclusion:** in-patient stay in the orthopedic and geriatric rehabilitation clinic; age ≥ 50 years; able to score ≥ 20 on the Mini Mental State Examination (MMSE); able to provide a signed informed consent; physically able to stand for at least 3 min without external support (self-report).  **Exclusion:** mobility or cognitive limitations or comorbidities which impair the ability to use the training games and overall system; conservatively treated osteoporotic fractures; previous or current major psychiatric illness (e.g., schizophrenia, bipolar disorder, recurrent major depression episodes); history of drugs or alcohol abuse; terminal illness; severe visual (e.g., especially achromatopsia) and auditory impairments; insufficient knowledge of German to understand the instructions/games  (39) EX:73.0 years; CO:72.2 years | No adverse events occurred during the training sessions (and not during the pre- and post-assessments) | NO/NO |  |
| **5.** Patrick (2023) Front Aging Neurosci (Switzerland) | to evaluate the feasibility, system usability, acceptance and effects of the “Brain-IT” project | a pilot RCT (conducted between July 2021 and June 2022) | On average, we recruited 2.2 participants per month, and 35.3% of the individuals contacted were included. The intervention group had an attrition rate of 20% and mean adherence and compliance rates of 85.0 and 84.1%, respectively. The mean system usability score, measured with the system usability scale, was 71.7. High levels of exergame enjoyment, an increase in exergame enjoyment, and internalization of training motivation with large effect sizes (p = 0.03, r = 0.75 and p = 0.03, r = 0.74, respectively), as well as acceptable perceived usefulness, were observed | **Inclusion:** • (1 = mNCD) clinical diagnosis of “mild neurocognitive disorder” according to the International Classification of Diseases 11th Revision (ICD-XI); or the latest Diagnostic and Statistical Manual of Mental Disorders 5th Edition (DSM-5); OR (2 = sMCI) individuals screened for mild cognitive impairment (sMCI) according to the following criteria: (a) informant (i.e., healthcare professionals)-based suspicion of mild cognitive impairment (MCI) confirmed by (b) an objective screening of MCI based on the German Version of the Quick Mild Cognitive Impairment Screen (Qmci) with (b1) a recommended cutoff score for cognitive impairment (MCI or dementia) of < 62/100, while (b2) not falling below the cutoff score for dementia (i.e., < 45/100), while (c) activities of daily living remain intact (judged by the referring healthcare professionals); • Fully vaccinated against coronavirus (SARS-CoV-2) with a Swiss Federal Office of Public Health (FOPH)-approved vaccine; • German speaking; • age ≥ 50 years; • able to stand for at least 10 min without assistance  **Exclusion:** • Mobility impairments (i.e., gait and balance) that prevent experiment participation; • Presence of additional, clinically relevant (i.e., acute and/or symptomatic) neurological disorders (i.e., epilepsy, stroke, multiple sclerosis, Parkinson's disease, brain tumors, or traumatic disorders of the nervous system);• Presence of any other unstable or uncontrolled diseases (e.g., uncontrolled high blood pressure, progressing or terminal cancer);  **Additional COVID-19-specific exclusion criteria:** •Coronavirus Disease 2019 (COVID-19) specific risk factors (according to the Swiss FOPH) were additional exclusion criteria. In the case of COVID-19-specific exclusion criteria, participation in the study was only allowed when the participants' treating physician provided written informed consent allowing participation in the study despite the presence of COVID-19-specific exclusion criteria. COVID-19-specific exclusion criteria included; • High blood pressure (self-reported; systolic ≥140 mmHg and/or Diastolic ≥90 mmHg); • Chronic respiratory condition; • Uncontrolled type 2 Diabetes; • Condition or therapy that weakens the immune system; • Unstable cardiovascular disease; • Cancer (present and/or under treatment); • Serious obesity (body mass index ≥40 kg/m2)  (16) EX:79.9 years; CO:73.7 years | Three minor adverse events (falls in participants’ homes with bruises, but no more serious injuries) were recorded, all of which occurred in the intervention group (in two different participants, one of whom has mild frontotemporal NCD). All AEs were unrelated to the “Brain-IT” training | NO/YES |  |
| **6.** Pavel (2019) J Stroke Cerebrovasc Dis (Israel) | to investigate the feasibility of a VR S-MC with treadmill walking in chronic poststroke survivors | a RCT (-) | The adherence to the intervention was 100% for both groups, and there was no dropout during the study | **Inclusion:** (1) hemiplegia after a stroke for at least 1 year since the incident; (2) age range 40-80 years old; (3) not taking medication or with unchanged medication throughout the past half year or longer; (4) using an ankle-foot orthosis or having no splint at all; (5) and can do a regular walk of 10 m without and with single point stick.  **Exclusion:** (1) major cardiac problems; (2) a score of less than 25 in the Mini-Mental test; (3) fractures or severe orthopedic limitations that do not allow for training, and which occurred over the last 6 months; and (4) more than 3 falls in the year prior to participating in the study  (22) EX: (66 ± 8.6) years; CO: (64.4 ± 9.4) years | No adverse events were reported throughout the study period | NO/NO |  |
| **7.** Lakshmi (2019) Neuro Rehabilitation (USA) | evaluated the efficacy of a S-MC exergame among people with chronic stroke | a RCT (-) | One participant each from the CMT and CT groups declined to participate in the training. All individuals in the CMT group completed the training, but two CMT participants were excluded during analysis due to an error during data collection, and one participant from the CT group refused to perform the Slip-Perturbation test | **Inclusion:** Participants with onset of hemiparetic cortical stroke greater than six months ago, without any presence of aphasia and with diagnosis confirmed by their physician. Participants were required to be able to stand independently for at least five minutes without the use of an assistive device or physical assistance and needed to be able to follow instructions in English.  **Exclusion:** the Mini-Mental State Examination (MMSE) was used to determine the presence of cognitive deficits, and participants with scores <25/30 were excluded as lower scores on this scale are often correlated with dementia. Heel bone density scan was measured using the Lunar Achilles Insight, and individuals with a T-score less than – 2.0 were excluded as they were classified as osteopenia or osteoporotic. They were also excluded if they self-reported any metal implants due to orthopedic conditions or any other neurological disorders such as Parkinson’s disease, Alzheimer’s disease, vestibular deficits, peripheral neuropathy, or unstable epilepsy  (25) EX: (57,5±8,04) years; CO: (61±4.6) years | No adverse events were reported throughout the study period | NO/NO |  |
| **8.**Azusa (2023) Games Health J (Japan) | examined the effects of S-MC exergaming in healthy older adults | a RCT (-) | One female in the control group withdrew 4 weeks after starting the experiment for reasons unrelated to the study | **Inclusion:** community-dwelling older adults (14 males and 11 females, age 71–81 years), healthy enough to visit the laboratory on their own by car, bicycle, or on foot, and had no history of fall within the last year  (24) EX: (76.0 ± 3.3) years; CO: (74.9 ± 2.8) years | No adverse events were reported throughout the study period | NO/NO |  |
| **9.**Ying-Yi (2019) Front Aging Neurosci (Taiwan, China) | to assess the effects of a VR-based S-MC in older adults with MCI | a RCT (-) | Three participants in the VR group and five participants in the CPC group dropped out due to low motivation. A total of 34 participants (18 in the VR group and 16 in the CPC group) completed all the assessments | **Inclusion:** (1) aged 65 years and over; (2) able to walk more than 10 m without walking aids; (3) had a Montreal Cognitive Assessment (MoCA) score lower than 26 (Tsai et al., 2012); (4) had self-reported memory complaints; and (5) had the ability to perform ADLs  **Exclusion:** (1) dementia; (2) a history of malignant tumors with life expectancy less than 3 months; (3) the presence of an unstable neurological or orthopedic disease interfering with participation in the study; and (4) an education level less than 6 years (elementary school)  (42) EX:(75.5 ± 5.2) years; CO: (73.1 ± 6.8) years | No adverse events were reported throughout the study period | NO/NO |  |
| **10.**Daniel (2015) PLoS One (Australia) | investigated the effectiveness of an interactive S-MC in older adults | a RCT (participants were recruited between June-September 2013 and re-assessments were conducted between September 2013 and January 2014) | Eighty-one participants (90%) attended re-assessment | **Inclusion:** i) were aged 70 years or older, ii) lived independently, iii) were able to walk with or without a walking aid, iv) were able to step unassisted on a step pad (step size 25-30cm) and v) had no severe lower extremity pain  **Exclusion:** major cognitive impairment (Mini-Cog<3), diagnosis of a neuro-degenerative disease, color-blindness, corrected vision of less than 6/16 or an unstable health condition. All participants gave informed written consent prior to study participation  (90) (81.5±7.0) years | No falls or other adverse events related to the intervention were reported | NO/NO |  |
| **11**.Roberta (2023) Aging Clin Exp Res (Italy) | to verify the effects of a S-MC with feedback provision and variability of practice in older individuals | a RCT (-) | Participants’ compliance with the training programs was 100% except for one who had to withdraw from the study for orthopedic problems not deriving from the proposed exercises | **Inclusion:** age over 65 years, nonparticipation to regular vigorous intensity physical exercise, medically stable health condition verified through a medical history questionnaire ascertaining whether under medical treatments and absence of conditions potentially affecting the variables of interest to the study, or the participation to physical exercise classes (e.g., uncontrolled cardiac illness, history of cerebrovascular disease, severe lower limb arthritis, uncontrolled metabolic disease). Participants were then invited to attend the laboratory to undergo tests of functional mobility including balance and cognitive functions  (29) (74.8 ± 5.8) years | No adverse events were reported throughout the study period | NO/NO |  |
| **12.**Elisa (2022) Front Aging Neurosci (Italy) | explored the effect of the duration of training based on the S-MC interaction in subjects with PD | a RCT (-) | Training Compliance Participant dropouts during the training were similar between groups, approximately 12% in the 6-week group and 10% in  the 12-week group (Chi-square: p = 0.68), suggesting that the duration of training did not affect the participation of patients. In all, 11 patients (8 in the 6-week and 3 in the 12-week groups) abandoned the training due to personal reasons not related to training compliance | **Inclusion:** (1) diagnosis of idiopathic PD according to the United Kingdom Brain Bank criteria, (2) 2 or more falls in the previous 6 months, (3) aged 60–85 years, (4) Hoehn and Yahr (H&Y) stage II or III, (5) able to walk for 5 min unassisted, and (6) stable anti-Parkinsonian medication regimen for the past 1 month  **Exclusion:** (1) past history of neurological conditions other than PD, (2) Mini-Mental State Examination (MMSE) score <21, (3) psychiatric co-morbidity (e.g., major depressive disorder as in accordance with DSM IV criteria), (4) unstable medical condition in the past 6 months, and (5) unable to comply with the training or currently participating in another trial  (160) 6-Weeks Group: (73.84 ± 6.39) years; 12-Weeks Group: (74.09 ± 4.96) years | None of the participants sustained severe adverse events during the study | NO/NO |  |
| **13.**Patrick (2015) Clin Interv Aging (Switzerland) | to compare two variations and evaluate the effects of multicomponent S-MC in healthy elderly persons | a RCT (participants’ recruitment lasted from August 2012 until the end of September 2012, when pretests were performed. The training intervention lasted from October 2012 until the end of March 2013, with follow-up test in April 2014) | Of the 89 participants initially enrolled, 71 participants completed the 6-month training intervention (20.2% attrition) and were included in the analysis of the outcomes derived at pretest, 3-month, and 6-month tests. Dropouts were equally distributed between groups, and therefore, the final analyses were performed only in those individuals who completed the 6-month intervention | **Inclusion:** be older than 70 years, live independently or at residence facilities for the elderly, and sign informed consent. Participants had to be able to walk at least 20 m, with or without walking aids, for gait analysis. Residents of retirement homes classified 0, 1, or 2 within the Swiss classification system for health care requirements (BESA levels, German abbreviation for: Bewohner-Einstufungs- und Abrechnungssystem) could enroll in the study. Level 0 means the person does not need care or treatment and levels 1–2 mean that the person only needs little care or treatment. Judgment by their primary care physician was required in the case of acute or instable chronic diseases (eg, stroke and diabetes) and rapidly progressing or terminal illnesses before accepting a person for participation  **Exclusion:** Seniors diagnosed with Alzheimer’s disease, dementia, recent head injury, or a score <22 points on the Mini-Mental State Examination, which indicates cognitive impairment, were excluded  (89) Dance group:77.3 years; Memory group:78.5 years; Phys group:80.8 years | No adverse events were reported throughout the study period | NO/NO |  |
| **14.**Tom (2017) J Phys Ther Sci (Belgium) | investigated whether institutionalized older adults improved their performances by a VR S-MC | a RCT (-) | Two participants in the intervention group had to discontinue their training due to medical conditions not related to the program. The eight remaining participants were able to finish the six week intervention program without injuries or other inconvenience. Due to serious illness and hospitalization, one participant of the control group could not be retested, resulting in 17 subjects included for the analysis. Interviews with participants from the intervention group showed that they found the program useful for their concentration, memory and balance, according to the results of the IMI, which resulted in a high compliance. They scored the program as very interesting and pleasant to do and perceived their performance of the different exercises as good to very good | **Inclusion:** participants were able to walk 10 meters repeatedly with walking aid, lived for at least 3 months at the residential care center and suffered from mild cognitive impairment (MoCa<26)18)  **Exclusion:** participants who were still rehabilitating from a hospitalization (e.g., after a neurological disease or orthopedic surgery), had a diagnosis of dementia or major sensory or motor impairments of the upper or lower extremities which could interfere with the program were excluded  (20) (87.2 ± 5.96) years | No injuries or other inconvenience happened | NO/NO |  |
| **15.**Magdalena (2017) Eur J Ageing (Slovak Republic) | compared the effectiveness of S-MC, on cognitive functions and the transfer to ADL in older people with MCI | a RCT (conducted between June 2013 and March 2014) | In the experimental group, all of the included persons completed the programme. In the control group, two individuals did not complete the training programme due to respiratory disease. They developed acute symptoms in the middle of the running programme and they were unable to finish the programme | **Inclusion:** participants had to have mild cognitive impairment with the presence of a subjective mild decrease in memory and attention and had to be over the age of 65  **Exclusion:** participants were excluded if they had moderate or severe cognitive deficits (MMSE ≤23), major depressive or anxiety disorders (5 participants), cancer, significant visual or auditory damage, neurological diseases or brain injury or psychiatric disorders  (80) (67.07 ± 4.3) years | There were no adverse events or side effects recorded during or after the training | NO/NO |  |
| **16.** Ji-Su (2020) Healthcare (Basel) (Korea) | investigate the effects of VR C-MR, in older adults | a RCT (-) | Thirty-five of the 40 subjects completed the study; 5 subjects (VRCMR group [n = 2] and CCR group [n = 3]) dropped out due to refuse or poor participation rate | **Inclusion:** diagnosed with MCI through clinical examination by a neurologist, age > 65 years, Mini-Mental State Examination score > 16, no limitation in the upper extremity ranges of motion, fair grade on manual muscle testing of upper extremity, ability to grip objects with various forms (cylindrical, spherical, and power grip), ability to follow the study instructions, independence in daily activities, ability for adequate communication, no history of neurological disorders, including stroke, no history of visual perception deficits, and consent to participate actively.  **Exclusion:** unstable medical problems, history of psychiatric disorders, severe communication difficulties, problem with visual and auditory functions (e.g., color blindness, hearing impairment)  (40) VRCMR Group: (75.8 ± 8.5) years; CCR Group: (77.2 ± 7.2) years | No adverse events were reported throughout the study period | NO/NO |  |
| **17.**Bernardo (2023) Experimental Brain Research (Italy) | investigating the placebo effect on the DT cost of gait in older adults | a 2 × 2 within-between design RCT (-) | In the control group, one subject was excluded because of technical problems during data acquisition and one subject was excluded due to outlier values (2.5 × SD above or below the mean of the group) | **Inclusion:** Mini-Mental Status Examination score ≥ 24, and body mass index (BMI) < 29.99 kg/m2  **Exclusion:** the presence of any contraindication to tDCS, according to a safety questionnaire  (37) (66.57±5.65) years | No adverse events were reported throughout the study period | NO/NO |  |
| **18.**Jorge（2024）J Neuroeng Rehabil (Ecuador) | to assess the effectiveness of a cognitive-motor intervention based on immersive virtual reality (VR) that simulates an activity of daily living (ADL) on cognitive functions and its impact on depression and the ability to perform such activities in patients with MCI | a randomized controlled trial (RCT) with parallel groups, conducted at a single center and with single blinding | Five participants were excluded from the study, because they stopped attending the senior center (*n* = 4) and one participant lacked of motivation. In the experimental group three participants stopped attending the senior center | **Inclusion:** (1) age equal to or greater than 65 years; (2) attend a day care center without being institutionalized in a geriatric center; (3) maintain physical functionality (absence of any disability); (4) ability to understand the purpose of the study and voluntarily agree by signing the consent form; (5) present MCI; (6) have a Montreal Cognitive Assessment (MoCA) score between 19 and 25 points  **Exclusion:** (1) clinical diagnosis of dementia; (2) probable dementia, with a MoCA score equal to or less than 18 points; (3) neurological disorders, including stroke or traumatic brain injury (TBI) in the past 12 months; (4) history of mental or psychiatric disorders; (5) addiction to medication, drugs or alcohol; (6) difficulties using an HMD and operating the controller equipment; (7) medical conditions that could interfere with effective participation in and completion of the study; (8) visual and/or hearing impairment; (9) communication difficulties  （34）(76.38 ± 6.25) years | This approach also appears to be safe, as participants reported no adverse effects during the intervention | NO/NO |  |
| **19.**Marek (2024) J Clin Med (Poland) | to compare the effectiveness of a fully immersive virtual reality (VR) environment combined with a scope of dual-task activities regarding balance in older women | a randomised control trial with single-blinding of the sample | All subjects enrolled in the study completed the study | **Inclusion:** (1) age ≥ 75 years, (2) consent from an internist or geriatrician for each person to participate, (3) MMSE > 23, (4) Dizziness and Balance Screening Questionnaire score of 10 out of 12 possible, and (5) BBS > 38  **Exclusion:** (1) Eye diseases and dysfunctions preventing participation in the study, (2) Dizziness of neurological origin, (3) Functional limb shortening, (4) Dizziness and Balance Disorders Screening Questionnaire score below 10, (5) Parkinson’s disease, (6) Unstable cardiovascular disease, (7) History of stroke, (8) Lack of informed written consent to participate in the study protocol, (9) Use of orthopaedic equipment during walking, (10) Medical conditions preventing participation, (11) Concurrent participation in another improvement programme, and (12) No written consent to participate  （80）(76.73 ± 1.99) years | All subjects enrolled in the study completed the study without reporting any adverse effects | NO/NO |  |
| **20.** Rick (2024) J Med Internet Res (China) | examined the effects of VR motor-cognitive training (VRMCT) on global cognitive function, physical frailty, walking speed, visual short-term memory, inhibition of cognitive interference, and executive function in older people with cognitive frailty | a multicentered, assessor-blinded, 2-parallel-group, 1:1-allocation-ratio randomized controlled trial design | In the intervention group, 4.8% (7/146) of the participants did not receive the allocated intervention, defined as attending 0 training sessions. A total of 81.6% (119/146) of the participants attended ≥81% (13/16) of the expected sessions, 5.9% of the participants attended 50% (8/16) to 75% (12/16) of the expected sessions, and 12.5% of the participants attended 0% to 44% (7/16) of the expected sessions. In the control group, all 147 participants received usual care. After the completion of the intervention, in the intervention group, 14.4% (21/146) of the participants were lost to follow-up, and 23% (7/21) of these participants discontinued the intervention, defined as attending 0 training sessions. In the control group, 7.5% (11/147) of the participants were lost to follow-up. | **Inclusion:** (1) The participants were aged ≥60 years; (2) The participants were community dwelling, defined as not living in a long-term care facility in the previous 12 months; (3) The participants had cognitive frailty, defined as the coexistence of MCI and physical frailty without concurrent dementia. MCI is measured through a Montreal Cognitive Assessment (MoCA) score of <26/30. Physical frailty is measured as a Fried frailty phenotype (FFP) score of >0. The measurement of each phenotypic criterion followed the Fried method using local normative data. Exclusion of possible dementia was measured through a MoCA score of <19/30  **Exclusion:** (1) There was a confirmed diagnosis of dementia, defined as the documented diagnosis on the participants’ medical record; (2) The participants had restricted mobility, defined as a Modified Functional Ambulation Classification score of <7  （293）(74.5 ± 6.8) years | In all the VRMCT sessions (n=4874) conducted on participants in both intervention and waitlist control groups (including some preintervention trial sessions on some participants), the VR sickness symptoms reported by participants using the VRSQ ranged from 0.7% on symptoms of difficulty focusing and headache to 3% on the symptom of vertigo. Among all the reported VR sickness symptoms, the most reported frequency was “less than half” (ie, 82%-93%). Regarding the most reported VR sickness symptom (ie, vertigo), only participants in 1% of the total sessions reported to have experienced it “more than half” during the VR training. No participants reported severe untoward effects requiring medical treatment after the completion of the training. However, 0.5% of the total training sessions were reported to have technical issues related to the training system. | YES/YES |  |

**Abbreviations:** Alzheimer’s Disease-AD; Simultaneous Motor-Cognitive training-S-MC; Parkinson’s Disease-PD; Virtual Reality-VR; Cognitive Frailty-CF; Mild Cognitive Impairment-MCI; Activities of Daily Living-ADL; Dual-Task-DT; Freezing of Gait-FOG; Randomized Controlled Trial-RCT

**Table 1-table to be continued**

| **NO. of study** | **Arm-design/**  **intervention contents/sample size estimation** | **Intervention duration** | **Tech(s) applied** | **Outcome measure time points** | **Primary** **outcome measurements** | **Secondary outcome indicators** | **Results** |
| --- | --- | --- | --- | --- | --- | --- | --- |
| **1** | **2-arm**: **EX:** the TR group (a 6-week online real-time supervised motor-cognitive dual-task exercise treatment); **CO:** the control group (no physical or cognitive intervention was given to the control group for 6 weeks and was informed that they could receive the same treatment if they wished after the study was over)  The exercise program consisted of simple chair-based exercises. The session frequency, duration, and the number of exercises were changed gradually. To adapt the participants to the online program, the treatment started with 15-minute sessions 5 days per week, and gradually progressed to 40-minute sessions, 4 days per week. The progression of physical exercises was achieved by adding new exercises to the program, not by the number of repetitions or sets. Cognitive tasks were added to the physical exercises in accordance with the cognitive level of the participants. The difficulty of cognitive tasks was gradually increased each week. If the cognitive task was not accomplished, the previous week’s tasks were repeated. Participants in the TR group attended 25 exercise sessions over 6 weeks; make-up sessions were held within the same week for sessions that the participant could not attend. Caregivers in both the control group and the TR group were directed to participate in online information meetings about AD. All participants continued their routine pharmacologic treatment  The estimated sample size was determined with 90% power (α = .05) using the Power and Sample Size Program (PS Power) based on the minimal clinical significant change of MMSE (2.36) and SD (1.48) from a previous study [32]. The estimated number | 6-week | telerehabilitation | All outcome measures were performed at baseline and 6weeks (post-intervention) through a video conference by the same physical therapist | MMSE; 5XSST; TUG | static standing balance—the OLST; the Katz-ADL; the FIM; depressive symptoms among older adults—the GDS; anxiety levels —the BAS; the care burden—the ZCBS; coping with stress, being productive, and beneficial—the WEMWBS | there was no significant difference in the comparison of the primary outcome measures between the groups after treatment. In contrast, significant differences in all secondary outcome measures were observed in favor of the TR group (p < 0.05), except for the OLST, Katz ADL, and ZCBI scores (p > 0.05). In the TR group, the change in the mean of primary outcome measures (MMSE, TUG, and 5XSTS) from baseline to 6 weeks was greater than in the control group (p = 0.008, p =0.002, and p =0 .041, respectively). The mean changes of all secondary outcome measures were significant in the TR group (p < 0.05) compared with the control group, except for the ZCBI and OLST (p >0 .05). Secondary outcomes. The Shapiro–Wilk test showed that the data for all outcome measures except normal walking speed, maximal walking speed, dual-task walking speed, 5xStS and Go/No-Go average reaction time were not normally distributed. The Levene test reported non-significant differences between the groups and the time points of each outcome |
| **2** | **2-arm:** intervention group (exergame training on the Dividat Senso 5 times a week in addition to the conventional rehabilitation treatment); control group (conventional treatment only)  **EX**: The Dividat Senso is a certified class 1 medical device and has been specifically developed for clinical use in older adults. The Dividat exergames target specific cognitive and motor functions that are important for activities of daily living, such as executive and attentional function as well as balance and coordination. The games are played mainly by making steps in four directions (left, right, front, back), but also by shifting body weight. The training sessions were carried out five times a week. Each training session was planned to last around 15 min, during which participants played five to seven different games. All participants played the same games, with the difficulty level being raised after each week. Furthermore, the training software (DividatPlay) contains an algorithm that adapts the difficulty automatically and in real-time to the performance of a participant. In case of (1) low performance in a game; (2) subjective perception of the patient that the game is too difficult or too easy; (3) and evaluation by the local investigator, the training program could be slightly adjusted by the local study investigator. This ensures personalized training with an adequate training stimulus  **CO:** underwent the conventional rehabilitation program and had no training on the Dividat Senso. At the Rehabilitation Clinic Zihlschlacht, the standard procedure of the first week with respect to cognitive–motor therapies consists of 1 × neuro-psychological assessment, 2 × occupational therapy, 2 × fine motor skills therapy, 2 × PD group therapy, 3–4 × individual physiotherapy and daily ergometer cycling. Subsequently, every participant got a program that was individually adjusted to their needs  A sample size calculation showed that that in order to correctly reject the null hypothesis with a power of 91%, a total sample size of 16 participants was required | was adjusted to the length of stay of the patients in the clinic and was usually between 2 to 4 weeks | Dividat Senso | before and after the intervention, pre- and post-measurements | feasibility (adherence rate, attrition rate, occurrence of adverse events, system usability scale (SUS), and NASA TLX score) | Various cognitive (Go/No-Go test, reaction time test (RTT), color word interference test (D-KEFS) and Trail Making Test A and B (TMT)) and motor (preferred gait speed, maximum gait speed, dual-task gait speed, Short Physical Performance Battery (SPPB), Timed Up and Go (TUG) and 5 times Sit-to-Stand (5xStS)) tests were conducted before and after the intervention phase in order to determine training effects | The mean NASA TLX value was 56.2 and the mean value of the SUS was 76.7. Significant time–group interaction effects were observed for the 5xStS, the SPPB, the RTT, the Go/No-Go test and the D-KEFS 2 |
| **3** | **2-arm:** the VR partially simultaneous motor-cognitive training (ie, the experimental group); the non-VR sequential motor-cognitive training (ie, the control group); while keeping the twice-weekly sessions at 30 minutes each  **EX:** cognitive training was delivered through a serious video game, which included training in eight daily living tasks commonly performed by older people in Hong Kong, and were arranged in eight progressive stages. They included orientation, finding a bus stop, reporting lost items, finding a supermarket, grocery shopping, cooking, finding a travel hotspot, and bird watching. These tasks tax cognitive functions such as visuospatial (eg, wayfinding), calculation (eg, settling payment), memory (eg, recalling items while grocery shopping), reaction time (eg, flipping eggs when cooking), and attention (eg, getting off a bus). Each week featured tasks involving two levels of difficulty in terms of cognitive demands (eg, more distractors, a higher complexity of items to be memorized, a shorter time for reaction). If the participant could complete the lower level in the first session in the week, they could proceed to the higher level in the second session of the same week. Motor training was provided by cycling on an ergometer, which allows cycling resistance adjustments to be made to increase the effort of cycling. The training system requires the participants to travel in the virtual world of the game through cycling on the ergometer while simultaneously participating in the cognitively demanding daily-living tasks. Tailoring of the training was allowed. The level of difficulty, cycling resistance, and target cycling distance could be adjusted according to the participant’s preference and previous cycling performance. The settings were determined at the beginning of each training session. Both the interventionist and participant mutually agreed on the settings before each session of training started. This design did not demand all tasks to tax motor and cognitive function simultaneously, the majority of the tasks demanded motor-cognitive functions simultaneously (6/8, 75%).  **CO:** the intervention for the control group involved providing motor and cognitive training sequentially on a non-VR platform. Materials included a tablet computer (Microsoft Surface Pro 7) and an under-desk ergometer (DeskCycle 2). Cognitive training was provided by a series of cognitive games performed on a tablet computer. The cognitive games included (1) Card Pairs (ie, attention), (2) Mind Game Double Memory (ie, memory), (3) Flashcard Maths (ie, calculation), and (4) Mind Game Double Connect the dots (ie, visuospatial). Participants were asked to cycle on the ergometer to complete the motor training. The four games were all planned by level of difficulty according to the demand on the cognitive load (eg, more distractors, a higher complexity of items to be memorized, a shorter time for reaction). The motor and cognitive training were provided sequentially (ie, cognitive training followed by motor training). The intervention lasted for 8 weeks with 2 sessions per week. The dose was comparable to that in the intervention group. Each training session lasted for 30 minutes, which included tablet-based cognitive training for 15 minutes followed by motor training for 15 minutes. Two cognitive games were offered to the participants in each session. The participants continued the game levels from the previous session. During the cycling segment of the session, the participants were not allowed to do anything other than cycling (eg, watch TV, browse on their smartphone)  NO | 8 weeks | a training system included an immersive VR platform with a head-mounted VR display, headphones and wireless handheld controllers (HTC VIVE Focus Plus), under-desk ergometer with adjustable cycling resistance  (DeskCycle 2), motion sensor, wrist-worn heart rate sensor (Polar OH1), and video game developed by the team | Demographic data were collected at baseline (T0) and outcome data were collected at both baseline (T0) and the week after completion of the intervention (T1) | adherence, adverse outcomes, and successful learning.  MoCA; FFP;  TUG | NO | At baseline, the median Montreal Cognitive Assessment score was 20.0 (IQR 4.0). No significant between-group differences were found in baseline characteristics except in the number of chronic illnesses (P=0.04). At postintervention, the intervention group (Z=–2.67, P=0.01) showed a significantly larger improvement in cognitive function than the control group (Z=–1.19, P=0.24). The reduction in physical frailty in the intervention group (Z=–1.73, P=0.08) was similar to that in the control group (Z=–1.89, P=0.06). Improvement in walking speed based on the Timed Up-and-Go test was moderate in the intervention group (Z=–0.16, P=0.11) and greater in the control group (Z=–2.52, P=0.01) |
| **4** | **2-arm:** intervention group (exergame-training using the Dividat Senso in addition to the conventional rehabilitation treatment); control group (conventional treatment only)  **EX**: The Dividat Senso is a device consisting ofa pressure-sensitive platform which records movement produced forces. The platform includes 20 sensors (strain gauges), five vibration motors and an LED control. It is certified as a medical device class 1 and was specifically developed for clinical use. The Dividat Senso is connected to a computer and a screen on which the stimuli appear. The Dividat exergames were used which specifically target cognitive and motor functions required for activities of daily living such as executive and attentional functions and balance and coordination. The games are played by making steps in four directions (front, right, left, back) and body weight shifting. Training sessions were executed on 5 days per week and each session lasted between 10 and 15 min. During each session, the participants played between six and seven different exergames each lasting between 2 and 3 min. The participants played the same composition of games for five training sessions. After every five training sessions, new, more challenging games were introduced to the training plan. To ensure adequate training progression, personalization of the training plan was achieved on the one hand by the training software (DividatPlay), which contains an algorithm that enabled automatic, real-time adaptation of the difficulty of a training to the level of an individual participant. On the other hand, the therapist/trainer adapted the training plan (i.e., substituted single games) in case of insufficient or excessive difficulty as measured by two criteria: too low performance in a game, subjective report of the patient that the game is too difficult or too easy  **CO:** The patients of the control group followed the standard rehabilitation plan offered by the clinic. For each week this usually included: 3× 30 min physiotherapy, 5× 30 min group therapy (knee- / hip- or back-specific group / otago-group therapy for upper extremities), 3× 30 min walking groups (only in patients admitted for issues in the lower extremities), 3× 45 min group therapy (mindfulness therapy, medical training therapy, activating groups)  To ensure sufficient power, a sample size calculation was performed. Sample size calculation suggested that a total sample size of 16 participants would offer a power of 91% to correctly reject the null hypothesis | the duration of the intervention period was adjusted to the duration of each participant’s stay at the rehabilitation clinic lasting between 2 and 3 weeks | Dividat Senso | all secondary outcome measures were assessed pre- and post-intervention | adherence rate, attrition rate, occurrence of adverse events, the SUS and NASA-TLX score | secondary outcomes included measures of physical and cognitive functioning such as comfortable walking speed, maximal walking speed, dual task walking speed, SPPB, TUG, D- KEFS, TMT, Go/No-Go test and SRTT | The mean SUS score was 83.6 and the mean NASA-TLX score 45.5. Significant time-group interaction effects were found for the dual task walking speed, the Go/No-Go test and SRTT |
| **5** | **2-arm:** the intervention group (a 12-week training according to the “Brain-IT” training concept in addition to usual care); the control group (usual care as provided by (memory) clinics where the participants were recruited)  **EX:** a 12-week training according to the “Brain-IT” training concept consists of an individually adapted multi-domain exergame-based simultaneous motor–cognitive training with incorporated cognitive tasks combined with HRV-guided resonance breathing. It is adopted with a deficit-oriented focus on the neurocognitive domains of (1) learning and memory, (2) executive function, (3) complex attention, and (4) visuospatial skills. Each participant was instructed to train ≥5x/week for ≥21 min per session resulting in a weekly exercise volume of ≥105 min  **CO:** usual care of mNCD typically includes treating medical conditions other than mNCD (e.g., diabetes mellitus and depressive symptoms), controlling comorbidities (e.g., hypertension and obesity), and managing risk factors (e.g., smoking habits and physical and cognitive inactivity). With this regard, usual care may include medication, recommendations for changing lifestyle habits (e.g., living a cognitively, physically, and socially active life), physiotherapy to treat specific health problems such as back pain or mobility problems, occupational therapy, or day clinic visits. Usual care is highly individual, which varies between (memory) clinics where participants are recruited, and it is unclear whether participants comply with the recommendations of their clinicians  The sample size was justified based on the rules of thumb of Julious (2005), who recommended a minimum sample size of 12 per group for pilot or feasibility studies (Julious, 2005). As described in the Section Trial design and study setting, the focus of this study was on investigating the primary outcomes in the group receiving our new “Brain-IT” training. Considering the 2:1 allocation ratio, we targeted a sample size of 12 for the intervention and six for the control group, leading to a total sample size of n = 18. To ensure an adequate number of participants in the study, a safety margin for an attrition rate of up to 40% (criterion for orange light; see the Section Feasibility) was chosen. Based on these considerations, we aimed to recruit a total of 18–25 participants | 12-week | Dividat Senso | Pre- and post-measurements conducted within 2 weeks before starting and after completing the intervention period. All measurements were led by two investigators of our research team trained in the application of the measurement techniques and protocols | Feasibility included recruitment, adherence, compliance, and attrition；Usability— the validated German version of SUS ；User acceptance of the newly developed exergame-exergame enjoyment, training motivation, and perceived usefulness | As secondary outcomes, changes in global cognitive functioning and key neurocognitive domains of (1) learning and memory, (2) complex attention, (3) executive function, and (4) visuospatial skills, as well as resting-state cortical activity, spatiotemporal parameters of gait, psychosocial factors [i.e., QoL, and levels of depression, anxiety, stress], and cardiac vagal modulation [vm-HRV] were assessed | In table 1, “Feasibility, acceptability, and adherence”.section |
| **6** | **2-arm:** an experimental group (Dual-Task Walking -DTW); a control group (Single-Task Treadmill Walking -TMW)  **EX:** Each training session began with 8 minutes of warm-up that included mobilization and flexibility exercises and a 2-minute walk around the gym. Then, for safety reasons, the participants were attached to a harness. The participants began to walk slowly on the treadmill and continued for 3 minutes. In the phases that followed, the participants walked at the same speed while training with 3 VR games: (1) the ball game—participants were required to strike the virtual balls with their upper extremity, approaching them from different targets. In later stages of the game distractions were added, such as virtual shoes approaching randomly from different directions, which had to be avoided; (2) reactive boxing-virtual boxes appeared randomly and at fixed distances on both sides of the screen. The participant needed to touch the virtual box within a specified period of time; (3)cleaning windows—the participants were required to clean a series of windows as quickly as possible by wiping off the virtual dirt that covered the window  **CO**: In this intervention, participants performed the same warm-up routine as in the DTW, including mobilization, flexibility, and walking around the gym, and then continued to walk for another 20 minutes on the treadmill at a speed that was equivalent to the intensity of 60%-70% of their heart rate reserve (calculated by the Karvonen method)  NO | twice per week for 4 weeks | The SeeMe system (Brontes Processing: Gliwice, Poland)—a projected video-capture VR system that works with a standard PC and a single, standard web video camera | Preintervention test—performed 1 week prior to the intervention period; Intervention period—the interventions included 8 treatment sessions carried out twice per week for 4 weeks; Postintervention test—performed on the day after the end of the intervention period; Follow-up test—performed 4 weeks after the end of the intervention period | 10 mW, TUG, the FRT, the LRT-L/R; the ABC scale, and the BBS | NO | (1) a significant interaction with medium ES favoring the intervention group was demonstrated between groups and time in 10 mW m/s (F = 3.43; P =0.04; hp 2 = 0.15). Posthoc pairwise comparisons across group revealed significant differences between the pre- and postperiods in the experimental group (FDR critical P = .037); (2) a significant interaction with medium ES favoring the intervention group was demonstrated between groups and time in number of steps completed during the 10 mW steps (F = 3.49; P = 0.04; hp 2 =0 .15). Posthoc pairwise comparisons across group revealed significant differences between the pre- and post-periods in the experimental group (FDR critical P = 0.031); (3) no significant interaction with medium ES was found across groups and time in the Timed Up and Go test (F = 1.53; P = 0.23; hp 2 = 0.07). Posthoc pairwise comparisons across group revealed significant differences between the pre- and postperiods in the experimental group (FDR crit- ical P = 0.05); (4) a significant interaction with large ES across groups and time favoring the intervention group was demonstrated in ABC (F = 9.0; P = 0.001; hp 2 = 0.31) |
| **7** | **2-arm:** cognitive-motor exergame training (CMT); conventional balance training (CT)  **EX:** Cognitive-motor exergame training (CMT) Participants played Wii Fit games in conjunction with performing cognitive tasks during all of the 20 sessions. Each session was divided into three sub-sessions, with each sub-session comprised of four Wii-fit games (Bubble Balance, Table Tilt, Tight-Rope Walking, Soccer Head) played for five minutes while also incorporating three different cognitive tasks. During the games, participants stood on a balance board which was designed to sense the symmetry of their body weight distribution. The research assistant repeatedly prompted participants to pay equal attention to both tasks simultaneously (Wii gaming and the cognitive tasks) when cues were provided. At the end of each game, scores were presented on the screen to provide immediate feedback on their performance. When participants aced the game for two consecutive sessions, they progressed to more complex games (Basic Run or Basic Step).Similarly, cognitive tasks performed in conjunction with the Wii-Fit games also increased in complexity or difficulty (e.g., starting with single digit addition, 5 + 3, and progressing to double-digit addition, 19 + 14, and then to multiplication)  **CO:** Conventional training (CT) Participants receiving CT underwent a set of customized balance training exercises for 90 minutes. The first 10 minutes were spent on regular warm-up stretching exercises such as arm movements, trunk twists, neck movements, and both sideways and forward lunges. The following 15 minutes were spent on functional strengthening exercises, including high stepping, lunges, and squats, and progressing to resistance training using TheraBands and weights. The next 35 minutes were spent on balance training exercises such as standing on a firm or compliant surface with eyes opened and closed for varying durations, sit-stand exercises, single-leg standing, maintaining upright posture sitting on a medicine ball, and reach outs and step-ups to initiate stepping responses. Lastly, treadmill walking was performed as endurance training for 10–15 minutes. As a progression, stair climbing and/or over ground walking while avoiding obstacles could be added depending on the ability of the individual  NO | 6 weeks | four Wii-fit games (Bubble Balance, Table Tilt, Tight-Rope Walking, Soccer Head) with participants stood on a balance board | the pre-test was conducted on the same day that the baseline was measured. The post-test was conducted one day after the completion of the intervention | The Limits of Stability (LOS) test was administered using the Balance Master (Computerized Dynamic Posturography, Equitest Neurocom); To determine performance on the reactive balance control, a single slip-perturbation caused by the forward movement of motorized treadmill belt was induced at 0.20 m/s for 0.41 seconds with an acceleration of 12 m/s; Changes in cognition were assessed using the Direct RT Empirisofttrademark software to assess working memory, attention, and information processing speed based on the participants’ answers for the LNS task; the SPT and the LOS test were performed along with the LNS task | the BBS, the Six-Minute Walk test was performed in a 30 m walkway, the TUG test, the scores in S-MC training in the Wii-balance games | Post-intervention, under DT reactive conditions, CMT group improved both motor and cognition, while the CT group improved motor alone. Under DT volitional conditions, motor performance improved only in CMT group |
| **8** | **2-arm:** a training group; a control group  **EX**: We programmed dual-tasking standing balance training using analog and video exergaming. For the analog exergaming, participants stood on either leg selected at random and held a 30 · 23cm plastic tray that had the four corners marked with 1.0 cm diameter colored circular sticker. The task was to control the tray so that a ping-pong ball would touch the corner target in a specific order. Participants were encouraged to control the ball accurately and complete a trial in the shortest possible time. If the ball fell out the tray or participants lost balance, that is, free leg touched the floor, the technician stopped the trial and a new trial started. With 2 minutes of seated rest between trials, participants performed three trials on each leg. the duration of balance training was ∼15 minutes per day, 2 days per week  **CO**: balance training  NO | 8 weeks, in total 16 sessions | The Balance Ski , Table Tilt , and Balance Bubble games were adjusted to individual level of game difficulty, performed on two legs, while standing on a plastic covered Wii Fit stable board | at baseline and after 8 weeks of balance training or no training | evaluated participants' single-task static balance using single-leg standing time, evaluated participants' dynamic balance using the functional reach test and evaluated participants' physical ability by walking speed and leg muscle strength | NO | There was a group by time interaction in one-leg standing time (F = 14.5, P < 0.001, ηp2 = 0.40). Post-hoc analysis showed that the control group decreased standing time by ∼7 seconds after 8 weeks (P = 0.003) and the training group increased standing time by ∼25 seconds (P < 0.001, Table 1). Table 1 shows that there was a group by time interaction in functional reach distance (F = 10.5, P = 0.002, ηp2 = 0.32). There was no change in the control group after 8 weeks (P = 0.28). Functional reach distance increased after balance training by ∼7 cm in the training group (P = 0.001).There was no interaction and main effects in habitual (group by time: F = 0.003, P = 0.96, group: F = 0.25, P = 0.63, and time: F = 1.23, P = 0.28) and maximal walking speed (group by time: F = 2.18, P = 0.15, group: F = 0.54, P = 0.47, and time: F = 1.32, P = 0.26). There was no interaction in beam walking distance on a 4-cm-wide beam (group by time by task: F = 1.30, P = 0.26, group by time: F = 0.20, P = 0.66, time by task: F = 0.28, P = 0.60, and group by task: F = 0.017, P = 0.90).There was no interaction in beam walking distance on a 4-cm-wide beam (group by time by task: F = 1.30, P = 0.26, group by time: F = 0.20, P = 0.66, time by task: F = 0.28, P = 0.60, and group by task: F = 0.017, P = 0.90) |
| **9** | **2-arm:** the VR raining group, and the combined physical and cognitive training CPC) group**,** both group participated in a 60-min, training each visit, three times a week  **EX:** We used the Kinect system (Microsoft Corporation, Redmond, WA, USA) to capture the limb motions and create a full-body 3D virtual map. The physical elements of the VR training were developed by the well-established and widely used Tano and LongGood programs. We adopted programs, including a simplified 24-form Yang-style Tai Chi, resistance exercise, aerobic exercise, and functional tasks in the forms of window cleaning, goldfish scooping and other tasks relevant to daily activities, to improve upper and lower extremity balance, stability, strength and endurance. In the VR context, participants would imitate the virtual character and adjust their movements based on the simultaneous visual and auditory feedback. VR-Based Cognitive Training Program The cognitive training required wearing the VR glasses on their heads with a motor controller in both hands to execute the training tasks. Our laboratory invented most of the cognitive training VR games, while others were derived from the ‘‘Job Simulator’’ software developed by Owlchemy Labs. The concept of the cognitive programs was inspired by simulated IADL tasks. For example, in the taking mass rapid transit (MRT) game, participants took the MRT in a familiar VR context where station gates, ticket vending machines, and ATMs were located in the usual places. To complete the task, a participant needed to be aware of their present location and the designated stations. They also needed to gather enough coins based on the fare chart to obtain a ticket. In the store finder game, a big red cross sign appeared as an indicator when something was going wrong. A participant needed to virtually walk to the store noted on a map in less than 3 min. If the participants failed to get closer to the targeted store in 2 min, directional marks in red popped up to guide their way. In the kitchen chef game, a participant found herself/himself in a well-equipped kitchen surrounded by numerous utensils available for use to prepare an ordered dish. Once he or she was able to complete a simple meal, a more complicated dish requiring more ingredients and utensils to complete followed. The last game was convenience store clerk; participants were responsible for gathering items from the to-do list and checking them out. Some of the listed items were easy to find, while others could not be located as easily.  **CO:** Our CPC program contains both physical and cognitive elements of training. The physical training regimen comprised resistance, aerobic and balance exercises that meet the standards of the American College of Sports Medicine for seniors. Our physical training was set to reach 50%–75% of the maximal heart rate (calculated as 220- age) with the exertion perceived by the participants as ‘‘somewhat hard’’ (scored 13–14). Specifically, Therabands were applied to assist the training of both the upper and lower extremities during the resistance exercise. A series of whole-body aerobic exercises, for example, stepping while in the seated and standing positions, as well as on and off of a stool, was performed. Balance exercises included standing on a steady foam mat in various postures and walking forward and backward with eyes open and closed. Other functional tasks simulated ADLs and were designed to enhance motor performance and were integrated into the CPC program. Samples of functional tasks included asking participants to climb stairs, cross obstacles while reaching for objects, and turning and rising from a chair. In addition to the functional tasks, training that targeted cognitive abilities was also integrated into the physical training program. Training scenarios included walking while reciting poems, naming flowers and animals while crossing obstacles, solving math questions duringthe resistance training, drawing a circle in the air in the clockwise or counterclockwise direction with the right or left hand, respectively, and searching for the prefix and roots of a Chinese character at moments when they repetitively stand up from a chair  NO | 12 weeks | the Kinect system, VR glasses, the cognitive training VR games, and the “Job Simulatorsoftware | at baseline and after completing the 36 sessions | the TMT and the SCWT, has been used to assess the ability of inhibition in executive function;Gait performance was measured in three conditions: (1) walking at their preferred walking speed (single task); (2) walking while executing a serial subtraction by three task, starting from a randomized 3-digit number (e.g., 100, 97, 94…; cognitive dual task); and (3) walking while carrying a tray with glasses of water (motor dual task) | NO | Of the six within group p-values for the TMT, VR group shows two significant values (TMT-B. delta TMT). None of the outcomes of TMT were found to have group × time interactions except for the TMT-B (a borderline significant p = 0.032). Of the four within group p-values for SCWT, both VR and CPC are significant (SWCT-numbers, SWCT-time); neither interaction is significant. The results of the single and dual task gait performance are shown in Table 3. For single-task gait, both groups have two significant p-values of 12 within group p-values (VR group: gait speed, stride length; CPC group: gait speed, cadence) and no interaction for between groups. For motor dual task gait, VR group has two significant within group p-values (gait speed, stride length), CPC group has three significant within group p-values (gait speed, stride length, cadence) of the 12 within group p-values, and none of the six interactions are significant. For cognitive dual task, VR group has three significant p-values of the 12 within group p-values (gait speed, stride length, DTCs of cadence), CPC group has no significant within group p-value. None of the single and dual task gait outcomes were found to have group × time interactions except for the cognitive DTCs of cadence (a borderline significant p = 0.018) |
| **10** | **2-arm:** intervention groups; control groups. Participants were asked to play each game at least once during each session as many times as they wished with the recommended dose of three 20-minute sessions per week  **EX:** The intervention comprised four games: Stepper, StepMania, Trail-Stepping and Tetris. The basic action of all games entailed making well-timed and directed steps to achieve as many points as possible. In addition, each game also targeted specific cognitive functions associated with fall-risk in older people (Fig 1). We incorporated both, parallel (dual-tasking/multi-tasking) and serial (solve cognitive task before taking a step) processing ofstepping and cognition. Individuals therefore were required to maintain their balance under differing postural and cognitive conditions. Each game was played by using the arrows on the step pad, similar to using a keyboard. To ensure an equal number of steps for individuals playing a given game and to avoid multiple small steps, participants were required to return to the two stance panels after each step for all tasks. Participants received visual feedback during game play and after each game as a game score. Each game consisted of a range of levels, with the harder levels requiring higher cognitive capacity and physical effort to perform the tasks. Participants were free to choose which level to play but were encouraged to start the sessions where they had finished last session. They were instructed to progress to a higher level when they considered they were performing well at their current level or considered the game level was not sufficiently challenging, to ensure progression of training intensity. Participants could also return to a lower level if they considered a game level was too difficult  **CO:** People allocated to the CG were given a brochure about evidence-based information on various health-related topics, such as fall prevention, staying active, exercising at home, healthy eating, eyesight care, choosing footwear and mobility and walking aids ([www.activeandhealthy](http://www.activeandhealthy). nsw.gov.au). CG participants were asked to continue with their usual activities during the study period  Data from our previous study [16] was used to estimate the required sample size. We calculated that for an effect size ofF = 0.344, a two-sided significance level of5% and 80% power, a total sample size of70 participants was required to detect a difference in the Stroop Stepping Task between the intervention and control groups. We anticipated a drop-out of20% so aimed to recruit 84 people | 16 week | four games: Stepper, StepMania, Trail-Stepping and Tetris | at baseline and post intervention | the SST | (1)Processing speed was assessed with the letter-digit and simple and choice reaction time tests. (2)The TMT was used to assess attention and EF (set-shifting) (3)The Victoria Stroop task was used to measure executive control by response inhibition (4)Depressive symptoms were measured using the PHQ-9 (5)Adherence was measured using the recorded logs ofthe system-use | There were no improvements with respect to the Stroop Stepping Test (primary outcome) in the intervention group. Compared to the CG, the IG improved significantly in measures of processing speed, visuo-spatial ability and concern about falling. Significant interactions were observed for measures of EF and divided attention, indicating group differences varied for different levels of the covariate with larger improvements in IG participants with poorer baseline performance. The interaction for depression showed no change for the IG but an increase in the CG for those with low depressive symptoms at baseline. Additionally, low and high-adherer groups differed in their baseline performance and responded differently to the intervention. Compared to high adherers, low adherers improved more in processing speed and visual scanning while high adherers improved more in tasks related to EF |
| **11** | **2-arm:** experimental group; control group, both following the same motor−cognitive training for five weeks, twice weekly for 30 min, with provision of feedback in the EX group and no provision of feedback in the CO group  **EX:** Training consisted of twice weekly, 30 min gross−motor coordination exercises with variable practice conditions combined with stimulus–response cognitive tasks generated by an interactive device. receiving feedback during training. The practice of the motor tasks followed the “serial” between-skill model as each motor task was repeated twice during the same cognitive exercise before moving to the next. No “variable” within-skill model was applied to ensure safety and reduce stress to the participants. It was assumed that the complexity of the cognitive task would have required attentional resources and induced substantial mental stress to the subject increasing the risk of loss of balance control. Nevertheless, considering that motor complexity affects the relationship between exercise and cognition in motor training the level of difficulty of the motor task progressed every week and each level was repeated in the two-weekly session, therefore a total of four levels were programmed. Moreover, for two of the four exercises (exercise no 2 and 4) the motor task varied slightly within the same week; cognitive skills were performed every week in a serial order as exercises were repeated twice before switching to another skill in a structured, predictable order. Four blocks of cognitive exercises for different  **CO:** the same motor−cognitive training for five weeks, but no provision of feedback in the CON group  The present study is a 5-week intervention study. A priori power analysis for mixed ANOVA, within-between interaction to detect an effect size (f = 0.25), with power set at 90% and α = 0.05, resulted in a total sample size of 30 participants | five weeks | the portable system “Witty-SEM” | before and after a 5-week intervention | cognitive function—the MoCA; Static balance— was measured by asking participants to maintain three positions for a maximum of 30 s; Dynamic balance—using the Timed up and go test, walking speed was measured over a course of 7 m on a nonslip surface; Lower limb muscle power—through the 5 Times chair rise test; Lower limb muscle endurance— through the 30 s chair rise test | NO | Both groups improved static and dynamic balance (p < 0.05), walking speeds (p < 0.05), lower limb strength (p < 0.05) and cognitive functions with greater gains observed in the experimental group (p < 0.01) |
| **12** | **2-arm:** 6 weeks TT + VR training; 12 weeks of TT + VR training. Each session lasted approximately 45 min  **EX:** 12-weeks treadmill plus VR patients were required to walk on the treadmill while avoiding virtual obstacles projected on the screen. The virtual environment is comprised of enriched visual stimuli engaging several cognitive domains such as EF (e.g., decision-making and planning), attention (e.g., ignoring distractors on the way), working memory (e.g., navigation), and visual processing (e.g., timing of motor planned action)  **CO:** 6-weeks of treadmill training plus VR;  NO | 6-week group underwent 18 sessions of TT + VR training, and the 12-week group with 36 sessions | TT + VR system (V-TIME) | Clinical and instrumental evaluations were performed before (PRE), immediately after (POST), 1 month after (FU-1m) the training, and 6-month post-intervention (FU-6m) by a blinded assessor. Testing was carried out while patients were ON medication and at the same time of day for each subject | Gait performance— participants were asked to walk under the following three conditions each lasting 1 min；Cognitive functions — using a computerized neuropsychological test battery (NeuroTrax Corp., Medina, Modiin, Israel) and clinical scales；Falls—the fall rate for 6 months before and after training | Secondary outcomes included several gait variables related to fall risk: gait speed under UW and OB, gait speed variability, and stride length under all the conditions (UW, DT, and OB), as well as leading and trail feet clearance under OB (Bertoli et al., 2018). Secondary outcome measures included scores of the other domains within the NeuroTrax™ battery. And the FES-I score | statistical analysis neither reveal any significant time × group interaction nor main group effect (always p > 0.05) for all gait parameters considered. For EF, the primary outcome, we found a significant time × group interaction (p-adj = 0.066), and post hoc analysis revealed significant improvements (p-adj = 0.036) at post-evaluation only in the 12-week group. statistical analysis showed a significant change in the IR of falls over time (p = 0.003) for both groups with a strong trend for greater improvement in the 12-week group (p = 0.051) |
| **13** | **3-arm:** 1) virtual reality video game dancing (DANCE), 2) treadmill walking with simultaneous verbal memory training (MEMORY), or 3) treadmill walking(PHYS)  **EX:** Video game dancing The program DANCE included virtual reality video game dancing as a simultaneous cognitive–physical training. This training component combines an attention-demanding cognitive action with a simultaneous motor coordination aspect.  **CO:**①Treadmill memory training The program MEMORY comprised treadmill walking with verbal memory exercise as a simultaneous cognitive–physical training. Verbal memory training consisted of a computer-based serial position training that was presented on a computer screen in front of the treadmill, with a standard computer mouse as an input device.②Treadmill walking  The program PHYS included aerobic treadmill walking without any additional cognitive task and acted as a reference group with exclusive physical training components. Participants were instructed to walk or run at a constant pace  NO | 6 months | used two Impact Dance Platforms (Positive Gaming BV, Haarlem, the Netherlands) and created various levels of difficulty in step patterns and frequency with the StepMania Software, and the E-Prime 2.0 Professional software (Psychology Software Tools, Pittsburgh, PA, USA) was used to program the training | assessments were performed four times: pretraining, after 3 months, 6 months of training (post training), and at 1-year follow-up | Indicators in E-Prime 2.0 Professional software | Fall frequency was assessed retrospectively for the 6-month period prior to the intervention, after 3 months and 6 months of training, and 6 months and 12 months after the intervention.The SPPB was used to assess lower extremity functioning with a balance test, a 3 m-walk test, and a five chair-rises test. Functional aerobic endurance performance was measured with the6-MWT following the guidelines of the American Thoracic Society.The FES-I was applied as a measure of fear of falling, while symptoms of depression were recorded using the German version of the GDS | Gait analysis：Linear global time effect showed significant performance improvements in all intervention groups from pretest to 6-month test in 19 of the 20 gait variables (all P0.05, R2 from 0.008 to 0.118). In the DTC gait variables, the linear global time effect did not show any significant reductions (P-values from 0.069 to 0.96). (Q-Performance remained unchanged from 6 months to (R-follow-up test in 12 of the 20 gait variables, whereas per(S-formance in six variables decreased (statistical analyses (T-available in Table S6). The variables “velocity preferred-DT” (U-and “step-time preferred-DT” showed significant improve-(V-ments (F(2, 44) =7.10, P=0.011, two-tailed, r=0.37 and (W-F(2, 44) =9.36, P=0.004, two-tailed, r=0.42, respectively). (X-DTCs of walking were maintained after 1-year follow-up in (Y-four of the ten DTC gait variables and were reduced, signifi- (Z-cantly or with a trend, in the six other DTC variables. |
| **14** | **2-arm:** intervention group; control group  **EX:** The subjects assigned to the intervention group, followed a training program with the Bio Rescue (RM Ingenierie, France) combined with their standard of usual care. Training sessions were planned two times a week for six weeks. The duration of each session was gradually increased from 18 minutes in week 1 to 30 minutes in week 5. The BioRescue training was offered by a physical therapist. Participants were asked to stand on the platform located 1.0–1.5 m away from a 55-inch TV-flat screen. A training session consisted of a number of 3-minute exercises. In paper it describes the nine exercises which were used to train balance, weight bearing, memory, attention, and dual tasking. The degree of difficulty of each exercise was adjusted to the perceived skill level of each participant separately. If needed, the subjects could take a 90-second break for up to twice per session  **CO:** The subjects in the control group continued their standard of usual care in the nursing home if applicable  NO | six weeks | the BioRescue (RM Ingenierie, France) combined with their standard of usual care | - | Balance and gait performance— the Tinetti-POMA scale and the iTUGCognitive-motor dual tasking— the iTUG was combined with an extra visual task (iTUG+DT)；the Dutch version of theMoCA ; the motivation during the exercise program—the Dutch version of the IMI | - | No changes were detected over time for either group with regards to the Tinetti-POMA or the MoCA. The total time of the iTUG improved significantly after 6 weeks training in the intervention group (17.2 sec versus 15.8 sec, p=0.02). The turn-to-sit transition improved in the intervention group by almost a second (p=0.02), whereas the sit-to-stand transition or turn duration did not improve. However, the step-time before the turn decreased significantly in the intervention group (0.7 sec versus 0.5 sec, p=0.02). All older adults were able to perform the visual task while sitting. However, difficulties were experienced when it was combined with the iTUG. The iTUG + DT proved to be very challenging, as only 41.2% of participants remembered to sit at the chair at the end of the TUG in combination with the dual task. As a result, the total duration and the parameters from turn to sit of the iTUG + DT were not analyzed. Instead, supplemental analysis showed that only 37.5% of participants in the intervention group remembered to sit down at the end of the TUG + DT at baseline in comparison to 75% after the intervention. No changes were seen in the control group. Further, the BioRescue training did not seem to have any effect on gait or dual task performance during the TUG + DT as the percentage of errors nor gait parameters improved. Interviews with participants from the intervention group showed that they found the program useful for their concentration, memory and balance, according to the results of the IMI, which resulted in a high compliance. They scored the program as very interesting and pleasant to do and perceived their performance of the different exercises as good to very good. The emotions during the program are rated with the OERS and presented in Table 5A. Overall, sadness, anger and anxiety were almost never experienced during the training with the BioRescue. These emotions appeared only as a small reaction to the failure of an exercise. Especially alertness and pleasure were seen during the training. In comparison to the standard physical therapy in the residential care center, BioRescue exercises were associated with more pleasure in the two participants who received both therapies |
| **15** | **2-arm:** intervention groups; control group. Both groups—the control and intervention groups—received physical training as a foundation to which CogniPlus was added in the experimental group The frequency was 30 min daily lasting for 10 weeks. Our programme contained the following elements of exercise: Walking over obstacles, 5 min; Walking with a directional change, 6 min; Change of walking base, 6 min; Speed walking, 3 min; Walking with a load, 5 min; Walking up and down the stairs, 5 min. Both groups went through 30 min of daily physical training for 10 weeks. Participants completed 20 training sessions, two sessions during each week. Each type of cognitive sub-programme lasted for 5–10 min during a single session. During one session, only attention, short-term and long-term memory were trained; then at the following session, the executive functions and visual-motoric coordination were trained. All of the cognitive functions were trained during each week. Both groups underwent 30 min of daily physical training with balance components  **EX:** received physical training as a foundation to which CogniPlus was added in the experimental group. Probands in the experimental group performed the following exercises: Alert (both forms)—while driving a virtual car, probands were standing and changing their position from the left foot to the right foot and standing on their toes. Names—while learning names, the probands were repeatedly standing up from their chair and then sitting down on their chairs. Pland (Form 1 and 2)—while planning their virtual daily activities, like shopping, doctor visits and social life according to a priority timetable, the participants were alternately taking steps forward and backward, to the left and to the right.  **CO:** received physical training  NO | 10 weeks | CogniPlus | baseline and a follow-up after 10 weeks of training sessions | the AVLT; psychomotor and personal speed—Stroop test; attention, psychomotor speed and visual search capability—the TMT, Form A; an overview of mental speed, concentration of attention and space orientation—the DRT-II; eye coordination—the NHPT | the BADLS | In the mini mental state examination (MMSE) and auditory verbal learning test (AVLT), significant differences were noted in favour of the experimental group (p < 0.0001). The effect size was large. In the Stroop attention tasks, significant differences were observed in favour of the experimental group in personal tempo, perception factor (p < 0.002), and the effect size was medium. In number of errors in favour of the experimental group (p < 0.0001), the effect size was large. No significant differences were found between the groups in the assessment of the perception load score and the increased load score. With regard to psychomotor tempo, the disjunctive reaction time (DRT) showed significant differences, with an increased number of incorrect reactions in the control group (p < 0.005); the effect size was medium. No significant differences in the assessment of number of correct reactions between the groups were found. The trail making test (TMT) Form A showed significant differences in favour of the experimental group (p < 0.01). The effect size was medium. Visual-motoric coordination, as assessed by the nine hole peg test (NHPT) (performed with dominant hand), also showed significant differences in favour of the experimental group (p < 0.01). The effect size was medium. In the assessment of ADL by the Bristol activities of daily living scale (BADLS), significant differences were observed in favour of the experimental group (p < 0.0001), and the effect size was large. |
| **16** | **2-arm:** the VRCMR group; the CCR group, both sessions were performed 30 min per day, 5 days/week, for 6 weeks.  **EX:** Virtual reality-based cognitive–motor rehabilitation. (A) After entering the bathroom, personal hygiene; (B) driving; (C) door opening; (D), shampoo; (E) attachable handles in various forms; (F) virtual reality-based cognitive–motor rehabilitation using Motocog  **CO:** CCR was performed with tabletop activities, including puzzles, wood blocks, card play, stick construction activity, maze and pencil–paper with table activities. The selection and level of tasks and training programs were chosen by experienced occupational therapists to match the patient’s cognitive function.  The sample size was calculated using G-power 3.1.9.3 software (University of Dusseldorf Dusseldorf, Germany). The power and alpha levels were set at 0.80 and 0.05, respectively, and the effective size was set at 0.9. According to a prior analysis, each group required at least 16 subjects. Therefore, 20 participants in each group were enrolled, considering possible dropouts | 6 weeks | VRCMR was performed using the MOTOCOG system (Cybermedic Inc., Gwangju, Korea) | evaluations were performed immediately before the start of the intervention (pre-training) and after 6 weeks of intervention (post-training) | the MoCA, the TMT-A/B, and the DST-forward/backward; a 0 to 10 numeric rating self-report scale (NRSS) was used to assess the interest and motivation of the subjects during training | no | (1)Cognitive Function Evaluation Based on within-group comparisons (pre-training vs. post-training), the VRCMR group showed a statistically significant improvement in the MoCA, TMT-A, TMT-B, DST-forward, and DST-backward scores (p < 0.001, all). In contrast, the CCR group showed statistically significant improvement in the MoCA (p = 0.047), DST-forward (p = 0.029), and DST-backward (p = 0.008) scores but not in the TMT-A (p = 0.079) and TMT-B (p = 0.060) scores (Table 2) Based on the between-group post-training comparison, the VRCMR group showed greater improvement than the CCR group in the MoCA (p = 0.045), TMT-A (p = 0.039), TMT-B (p = 0.040), and DST-forward (p = 0.011) scores, but not in the DST-backward (p = 0.424) (Table 2).(2)Interest and Motivation Evaluation Using NRSS The average scores for interest and rehabilitation motivation were 6.07 and 7.14 points in NRSS, respectively, for the VRCMR group, and 3.64 and 3.50 points in NRSS, respectively, for the CCR group. Based on the between-group comparison, the subjects in the VRCMR group had significantly higher interest and rehabilitation motivation than those in the CCR group (p < 0.001, both) (Figure 3) |
| **17** | **2-arm:** Placebo, Control  **EX:** In the dual-task condition (DT), participants were asked to count aloud following a serial subtraction of 7 from a starting number while walking. The starting number was different in the pre- and post-test (either 300 or 500 with a counterbalance order across participants). Participants were asked to prioritize their attention to the countdown in order to be as accurate as possible at the cognitive task. Whenever a mistake was made, the experimenter revealed the correct number, and the participant was asked to continue the subtraction from the given number. EX: Participants were asked to walk barefoot at their self-selected and usual pace on a 7.92 m sensorized carpet (GAITRite System, CIR Systems, Sparta, NJ), which is considered as a valid and reliable tool to acquire spatial and spatiotemporal gait parameters (Bilney et al. 2003). The walking task was performed six times, turning around two cones without stopping (Fig. 2A). The task was performed in two conditions. In the single-task condition (ST), participants just performed six repetitions of the walking path  **CO:** The task was performed in two conditions. In the single-task condition (ST), participants just performed six repetitions of the walking path. In the dual-task condition (DT), participants were asked to count aloud following a serial subtraction of 7 from a starting number while walking.  A priori computation of the sample size was performed with G*Power 3.1 (Faul et al. 2007) for F tests, by considering ANOVA for repeated measures and within-between interactions with two groups (Placebo, Control) and two measurements (Pre-test, Post-test) for our main outcome measure (i.e., dual-task cost for gait). Given that there were no published data regarding the modulation of gait dual-task cost by means of the placebo effect, we assumed an anticipated effect size (f) of 0.25, which is considered as ‘medium’ according to the literature (Cohen 1988). Assuming an anticipated effect size of f = 0.25, a p-value (or type I error rate) equal to 0.05, power (1-β error probability) of 0.8, correlation among repeated measures of 0.5 and no sphericity correction ε of 1, the resulting sample size was 34 | 2 week | a 7.92 m sensorized carpet (GAITRite System, CIR Systems, Sparta, NJ) | pre-test and post-test | (1)Gait performance—was evaluated at the ST and DT by measuring speed, step length, stride speed, stride length and stance time;（2)Performance at the cognitive task—calculating the total number of subtractions and the number of errors normalized to one minute;(3)Subjective parameters—the VAS | NO | (1)Gait speed Overall, the dual-task cost was reduced at the post-test (11.64 ± 1.49%) compared to the pre-test (16.01 ± 1.98%) (Session, ­F(1,33) = 11.273, p = 0.002, ηp2 = 0.255). More interestingly, the interaction Session × Group was significant ­(F(1,33) = 5.315, p = 0.028, ηp2 = 0.139). Post-hoc comparisons showed that the placebo group had less dual-task cost at the post-test (8.61 ± 1.65%) compared to the pre-test (15.83 ± 2.69%; p < 0.001, d = 1.49), whereas no difference across sessions was found in the control group (p = 0.511). Moreover, while the two groups were comparable at the pre-test (p = 0.929), at the post-test the placebo group had less dual-task cost than the control group (14.85 ± 2.33%; p = 0.035, d = 0.74). (2)Step length Even for the step length, the dual-task cost was significantly lower at the post-test (5.58 ± 0.73%) than at the pre-test (8.50 ± 0.77%) (Session, ­F(1,33) = 25.787, p < 0.001, ηp2 = 0.439). The analysis also disclosed a significant interaction Session × Group ­(F(1,33) = 4.576, p = 0.040, ηp2 = 0.122). Again, post-hoc comparisons showed that the placebo group had less dual-task cost at the post-test (4.46 ± 0.90%) compared to the pre-test (8.56 ± 0.86%; p < 0.001, d = 1.82), whereas no statistically significant difference was found across sessions in the control group (p = 0.069). (3)Stride speed Analysis of stride speed confirmed a significant Session effect ­(F(1,33) = 16.557, p < 0.001, ηp2 = 0.334), because of less dual-task cost at the post-test (11.48 ± 1.43%) compared to the pre-test (15.98 ± 1.91%). Post-hoc comparisons of the significant interaction Session × Group ( ­F(1,33) = 4.274, p = 0.047, ηp2 = 0.115) disclosed less dual-task cost at the post-test (8.68 ± 1.66%) compared to the pre-test (15.37 ± 2.64%) for the placebo group (p < 0.001, d = 1.43), whereas no significant difference was found across session for the control group (p = 0.168). Finally, the dual-task cost was significantly low er in the placebo group than in the control group (14.45 ± 2.21%) at the post-test (p = 0.043, d = 0.71), indicating the positive impact of the placebo procedure in increasing strides velocity when a secondary cognitive task was performed simultaneously. (4)Stride length Analysis of stride length confirmed less dual-task cost at the post-test (5.59 ± 0.74%) than at the pre-test (8.45 ± 0.79%) (Session, ­(F(1,33) = 23.543, p < 0.001, ηp2 = 0.416). The analysis also revealed a significant interaction Session × Group (F ­(1,33) = 4.921, p = 0.034, ηp2 = 0.130). Even in this case, post-hoc comparisons indicated less dual-task cost at the post-test (4.40 ± 0.92%) compared to at the pre-test (8.51 ± 0.89%) specifically for the placebo group (p <0.001,d = 1.76), while no statistically significant difference was found across sessions in the control group (p = 0.096) (5)Stance The analysis of stance did not reveal any significant effect or interaction (for all, p > 0.12). (6）Performance at the cognitive task Analysis of the number of subtractions and number of errors did not reveal statistically significant effects (for all p > 0.115). These results indicate that performance at the cognitive task was stable throughout the experiment in both groups, suggesting that participants followed the instructions to perform the cognitive task accurately（7）Subjective parameters Perception of fatigability was not significantly different between groups (pre-test: U = 152.0, p = 0.97; post-test: U = 116.5, p = 0.22) nor between sessions (placebo: Z = − 1.50, p = 0.13; control: Z = − 0.15, p = 0.87). Expectation scores and tDCS efficacy scores in the placebo group were significantly above 0 (Z = − 2.32, p = 0.020, r = 0.52; Z = − 3.62, p < 0.001, r = 0.85, respectively), suggesting that the placebo procedure induced positive expectations and belief on the efficacy of tDCS |
| **18** | **2-arm: EX:** To stimulate the motor system, collective social interaction activities that included conversations between therapists and participants were carried out, promoting non-verbal language, the exchange of thoughts and jokes. Balance exercises were then performed such as walking in a straight line, placing one foot directly in front of the other, maintaining coordination. Participants were also instructed to focus on a fixed point ahead and maintain an upright posture to ensure stability. In addition, resistance exercises such as squats were performed, with an effort regulated according to the individual capabilities of each person. Participants were reminded to perform squats slowly, keeping their back straight and knees in line with their toes to avoid injury. Finally, a series of whole-body aerobic exercises were performed, such as brisk walking, an accessible exercise for older adults, going up and down stairs, and social dancing individually and in pairs. They were encouraged to maintain a constant rhythm and coordinate their movements with the rhythm of the music during social dancing. Subsequently, individual cognitive training was performed using an immersive VR-based system that simulates a task of searching for ingredients in a kitchen cupboard. It includes an application that uses VR to simulate an instrumental activity of daily life (iADL).  **CO:** The intervention for the control group was performed under the same conditions as the experimental group maintaining the same sequential design, since the participants of this group also participated in the motor training through social activities, balance, resistance and low intensity aerobic exercises. After that, the individual cognitive training consisted of a task similar to that of the experimental group, but without the use of VR  Both interventions were conducted twice a week for six weeks, and each session lasted approximately 40 min.  NO | 6-week | VR | The evaluations were carried out before the start of the motor training program based on VR or pencil and paper (baseline) and after the interventions. | MoCA-S; SGDS-S; IADL-S | NO | Between groups comparison did not reveal significant differences in either cognitive function or geriatric depression. The intragroup effect of cognitive function and geriatric depression was significant in both groups (*p* < 0.001), with large effect sizes. There was no statistically significant improvement in any of the groups when evaluating their performance in ADLs (control, *p* = 0.28; experimental, *p* = 0.46) as expected. The completion rate in the experimental group was higher (82.35%) compared to the control group (70.59%). Likewise, participants in the experimental group reached a higher level of difficulty in the application and needed less time to complete the task at each level. |
| **19** | **2-arm: EX:** A training plan for the group in dual-task and VR conditions was developed by the Author, based on the study by Liu et al., involving dual-task motor exercises (1) walking with putting a ball between the hands, (2) walking with tossing a ball, and cognitive exercises (3) talking while walking, (4) walking whilst adding up numbers, (5) walking whilst subtracting numbers, (6) walking whilst repeating phrases, (7) walking whilst reciting a word chain, and (8) walking whilst identifying objects. In the first training session, questions required short answers, e.g., yes/no (Did you have breakfast today?) and in the following sessions, subjects were required to answer in the form of complex sentences (What did you have for breakfast today?).  **CO:** The training comprised both the sitting and standing positions, accompanied by the use of leg weight cuffs. After the warm-up, subjects performed a series of consecutive specific exercises (balance, flexibility and resistance training), which were rounded off with some stretching  The warm-up time was approximately 5 min, the specific training 50 min, and stretching 5 min. Training took place 3 times in each one of 6 consecutive weeks (total of 18 sessions, with 2 absences admissible)  The sample size was calculated using the Online Sample Size Calculator | 6-week | VR | Baseline; Six weeks after the commencement of the study (i.e., the conclusion of the intervention), Nine weeks after the commencement of the study (i.e., three weeks after the conclusion of the intervention) | MMSE; BBS; TUG; TUG COG; TUG MAN; SLS | NO | Six weeks after the commencement of the study (i.e., the conclusion of the intervention), the VR + DT group scored better on the TUG MAN, TUG COG, SLS OP, and SLS CL tests compared to the OTAGO group. The OTAGO group scored significantly better on TUG (11.35 s vs. 12.60 s, *p* < 0.001) and BBS. Nine weeks after the commencement of the study (i.e., three weeks after the conclusion of the intervention), the OTAGO group scored better in TUG (12.26 vs. 12.82, *p* = 0.01), TUG MAN, TUG COG and BBS, as compared to the VR + DT group. The VR + DT group performed better in the SLS OP and SLS CL tests. A significant improvement was observed in all test scores in both groups. There were improvements in TUG between pre- and post-intervention results in the OTAGO group (13.45 s vs. 11.35 s—difference 2.1 s 15.6% change, *p* < 0.001) and the VR + DT group (14.02 s vs. 12.60 s—difference 1.42 s 10.1% change, *p* = 0.001), in TUG MAN for the OTAGO group (13.72 s vs. 12.42 s—difference 1.3 s 9.4% change, *p* < 0.001) and the VR + DT group (13.23 s vs. 11.62 s—difference 1.61 s 12.1% change, *p* = 0.001), in TUG COG for the OTAGO group (16.16 s vs. 14.60 s—difference 1.56 s 9.6% change, *p* < 0.001) and the VR + DT group (17.02 s vs. 14.13 s—difference 2.89 s 17% change, *p* = 0.001), in the BBS for the OTAGO group (39.33 pts. vs. 42.58 pts.—difference 3.25 pts. 8.2% change, *p* < 0.001) and the VR + DT group (39.68 pts. vs. 41.88 pts.—difference 2.2 pts. 5.5% change, *p* = 0.001), in SLS OP for the OTAGO group (5.39 s vs. 7.90 s—difference 2.51 s 46.5% change, *p* = 0.001) and the VR + DT group (8.81 s vs. 9.87 s—difference 1.06 s 12% change, *p* = 0.01), and in SLS CL for the OTAGO group (1.40 s vs. 2.07 s—difference 0.67 s 47.9% change, *p* < 0.001) and the VR + DT group (1.52 s vs. 2.33 s—difference 0.81 s 53.2% change, *p* = 0.001). |
| **20** | **2-arm: EX:** the VR game contains 8 different themes: orientation, finding a bus stop, reporting lost items, finding a supermarket, grocery shopping, cooking, finding a travel hot spot, and bird watching. They mimic and gamify the problems faced and activities undertaken by older people regularly. In week 1, orientation sessions were provided to the participants aiming to teach them to master all the commands needed in the training (eg, cycling the ergometer to control the movement and speed in the virtual world and using the handheld controller to select and unselect items). In the remaining weeks, participants were instructed to navigate the virtual world by cycling on the motion sensor–connected ergometer and solve the gamified problems by exercising their cognitive capabilities in the virtual world. For example, in week 2, participants were expected to navigate the virtual world to find a designated bus stop to take a bus to a supermarket to continue the game. Participants were expected to memorize and recognize the visual cues in the virtual environment (eg, traffic lights and building names) to find the designated bus stop; while they were doing so, they would be exercising their visuospatial abilitie. As another example, in week 5, participants were expected to shop for food in the supermarket. Participants were expected to exercise their memory to recall the names of the food items needed for cooking, pay attention to find the image of the food items needed, and do the calculation to settle the payment  **CO:** Participants in this group received usual care. The research team did not provide any interventions to the participants in this group during the 8-week intervention period. Similarly to the participants in the intervention group, participants in the control group were also not restricted from enrolling on or engaging in any activities (eg, physical or social activities) provided by the community centers or organizations outside the community centers.  16 one-hour training sessions delivered twice per week for 8 weeks  We adopted a previous power analysis using the web-based software GLIMMPSE and used a general linear mixed model. We set the level of significance at 0.05, the power at 0.9, the number of repeated measures at 2 (ie, T0 and T1), the number of groups at 2 (ie, the intervention and control groups), and the allocation ratio between the 2 groups at 1:1. To estimate the effects, we referred to the interaction (ie, group × time) effect on global cognitive function (ie, primary outcome) observed in the pilot study with a highly identical design. The estimated sample size was 220. We assumed a dropout rate of 6%, as observed in the pilot study. The total sample size was expected to be at least 234 participants, with 117 in each group | 8-week | VR | These were measured at both baseline (ie, T0 at week 0) and the week immediately after the completion of the intervention (ie, T1 at week 9). | MoCA | FFP; TUG; DST | Cognitive function improved from T0 (mean MoCA score 21.12, SE 0.204) to T1 (mean MoCA score 22.10, SE 0.273) and reached the level of significance (within-group *P*<.001) in the intervention group but not in the control group (within-group *P*=.33). The extent of improvement in the intervention group was also significantly larger than that in the control group (group × time *P*=.03). Physical frailty decreased from T0 (mean FFP score 1.60, SE 0.060) to T1 (mean FFP score 1.29, SE 0.068) and reached the level of significance (within-group *P*<.001) in the intervention group but not in the control group (within-group *P*=.74), and the extent of reduction in the intervention group was also significantly larger than that in the control group (group × time *P*=.01). Executive function improved from T0 (mean TMT score 1.68, SE 0.054) to T1 (mean TMT score 1.51, SE 0.048) and reached the level of significance (within-group *P*=.01) in the intervention group but not in the control group (within-group *P*=.85). The extent of improvement in the intervention group was not but almost significantly larger than that in the control group (group × time *P*=.07). Walking speed, visual short-term memory, and inhibition of cognitive inference did not show more improvement in the intervention group compared to the control group. |

**Abbreviation:** the Mini Mental State Examination-MMSE; the One-leg Stance Test -OLST; the Katz Activities of Daily Living Scale-Katz-ADL; the Functional Independence Measurement-FIM; the Geriatric Depression Scale-Short Form-GDS; the Beck Anxiety Scale-BAS; the Zarit Caregiver Burden Scale-ZCBS; the Warwick-Edinburgh Mental Well-being Scale-WEMWBS; virtual reality-VR; the Montreal Cognitive Assessment-MoCA; the Fried Frailty Phenotype-FFP**;** the Timed Up and Go Test-TUG; the System Usability Scale- SUS; the Short Physical Performance Battery-SPPB, the Color-Word Interference test-D KEFS, the Trail Making test A and B-TMT/TMT-A/B, the Go/No-Go test and Step Reaction Time test-SRTT; quality of life-QoL; resting vagally mediated Heart Rate Variability- vm-HRV; the 10-m walking test-10 mW; the Functional Reach Test-FRT; the Lateral Reach Test Left/Right-LRT-L/R; the Activities-specific Balance Confidence scale-ABC; the Berg Balance Scale-BBS; the Letter-Number Sequencing task-LNS; the Slip-Perturbation test-SPT; the Limits of Stability test- LOS; the Stroop Color and Word Test-SCWT; the Stroop Stepping Test-SST; the nine-item Patient Health Questionnaire-PHQ-9; the 6-minute walk test (6-MWT); the Falls Efficacy Scale International-FES-I; the Performance-oriented Mobility Assessment scale- Tinetti-POMA; the instrumented Timed Up and Go test-iTUG; dual tasking-DT; the Intrinsic Motivation Inventory-IMI; the Auditory verbal learning test-AVLT; the Disjunctive reaction time-DRT-II; the Nine hole peg test-NHPT; the Bristol activities of daily living scale-BADLS; the Digit Span Test forward and backward-DST-forward/backward; a 0 to 10 numeric rating self-report scale-NRSS; the simulator sickness questionnaire-SSQ; the Unified Parkinson's Disease Rating Scale-UPDRS; the Technology Acceptance Model-TAM; the Beck Anxiety Inventory-BAI; the frontal assessment battery-FAB, the Unified Parkinson's Disease Rating Scale III-UPDRS III; the Freezing of gait questionnaire- FOG; the visual analogue scale-VAS; the Spanish version of the Short Form of Geriatric Depression Scale-SGDS-S; the Spanish version of the iADL scale-IADL-S; N-Back test; The Stroop test; The Timed Up and Go (TUG) test; CNS-VS- Central Nervous System Vital Signs; The WHO-5 Well-Being Index- WHO-5; EuroQol Five Dimensions Questionnaire- EQ-5D-5L; General Anxiety Disorder 7-item questionnaire-GAD-7; Timed up and go test cognitive- TUG COG; Timed up and go test manual- TUG MAN; A Single-leg Stance Test- SLS; the digit span test-DST
