# Supplementary material for: Technology-Assisted Motor-Cognitive Training Among Older Adults: Rapid Systematic Review of Randomized Controlled Trials
Source: JMIR Serious Games. 2025 Jun 3;13:e67250. doi: 10.2196/67250 (PMC12174886; doi:10.2196/67250)
Supplement: Multimedia Appendix 6 [file games_v13i1e67250_app6.docx]

|  | **1** | **2** | **3** | **4** | **5** | **6** | **7** | **8** | **9** | **10** | **11** | **12** | **13** | **14** | **15** | **16** | **17** | **18** | **19** | **20** |
| --- | --- | --- | --- | --- | --- | --- | --- | --- | --- | --- | --- | --- | --- | --- | --- | --- | --- | --- | --- | --- |
| **PHY** | TUG, 5xStS, Katz ADL, Functional Independence Measure | 5xStS, SPPB | FFP | - | - | BBS, FRT, LRT-L/R,10 m walking time, ABC | Performance under DT conditions on Limits of Stability (volitional) and Slip-Perturbation (reactive) tests | single leg standing time | single-task and motor DT gait performance measures | - | Static (the Romberg, Tandem tests, and One Foot) and dynamic balance (TUG), walking speeds (7m walking), lower limb strength (5xStS, the 30 s chair rise test) | - | step time at fast walking, gait, functional fitness, and fall frequency, gait variability at preferred walking speed, and SPPB | - | ADL | - | - | - | dual-task gait and single-leg standing, Single-leg Stance Test closed-eyed (SLS CL) | Fried frailty phenotype (FFP) |
| **COG** | MMSE | RTT, Go/No-Go, D-KEFS | MoCA | Go/No-Go, SRTT | - | - | letter-number sequencing | - | SCWT, TMT-B | processing speed, visuo-spatial ability | MoCA | executive function, visuospatial ability, and attention | - | concentration, memory, and balance | AVLT, Stroop,  DRT, TMT, NHPT | MoCA, TMT-A/B, DST-forward | - | MoCA | - | MoCA |
| **DT** | - | - | - | DT gait speed | - | - | - | - | DT gait performance measures, cognitive DT gait performance, DT costs (DTCs) of cadence | - | - | - | DT costs of step time variability at fast walking, gait variability in DT and DT costs at preferred walking speed | - | - | - | - | - | - | - |

**Abbreviation: PHY**-physical; **COG**-cognitive; **DT**-dual-task; **TUG**-the Timed Up and Go; **5xStS**-the Five Times Sit to Stand Test; **Katz ADL**-the Katz Activities of Daily Living; **SPPB**- the Short Physical Performance Battery; **FFP**- the Fried Frailty Phenotype; **BBS**- the Berg Balance Scale; **FRT**- the Functional Reach Test; **LRT-L/R**- the Lateral Reach Test Left/Right; **ABC**-the Activities-specific Balance Confidence scale; **MMSE**-the Mini-Mental State Examination; **RTT**-the Reaction Time Test; **D-KEFS**- the color word interference test; **MoCA**-the Montreal Cognitive Assessment; **SRTT-**Step Reaction Time test; **SCWT**-the Stroop Color and Word Test; **TMT-A/B**- the Trail Making Test A and B; **AVLT**—the Auditory Verbal Learning test; **DRT-**the Disjunctive Reaction Time; **NHPT**- the nine hole peg test; **DST-forward**-Digit Span Test forward; **SLS CL**-Single-leg Stance Test closed-eyed;

**Appendix 6-the summary of statistically significant outcome indicators in three domains**
